# Exclusion of HDAC1/2 complexes by oncogenic nuclear condensates

**DOI:** 10.1186/s12943-024-02002-1

**Published:** 2024-04-27

**Authors:** Junqi Kuang, Pengli Li, Ziwei Zhai, Yixin Fan, HuaiYuan Xu, Chengchen Zhao, Wei Li, Xiaoxi Li, Zechuan Liang, Tao Huang, Yue Qin, Huiru Gao, Zhaoyi Ma, Dong Liu, Guifa Zhong, Bo Wang, Jing Liu, Jin Wang, Micky D. Tortorella, Baojian Liao, Duanqing Pei

**Affiliations:** 1 Laboratory of Cell Fate Control, School of Life Sciences, Westlake University, Hangzhou, China; 2 Institute of Biology, Westlake Institute for Advanced Study, Hangzhou, China; 3 CAS Key Laboratory of Regenerative Biology, South China Institute for Stem Cell Biology and Regenerative Medicine, Guangzhou Institutes of Biomedicine and Health, Chinese Academy of Sciences, Guangzhou, China; 4 Guangdong Provincial Key Laboratory of Stem Cell and Regenerative Medicine, Guangzhou Institutes of Biomedicine and Health, Chinese Academy of Sciences, Guangzhou, China; 5 University of Chinese Academy of Sciences, Beijing, China; 6 Laboratory of Stem Cell and Regenerative Biology, the Sixth Affiliated Hospital of Guangzhou Medical University, Qingyuan People’s Hospital, Qingyuan, China; 7 Department of Musculoskeletal Oncology, Sun Yat-Sen University Cancer Center, Guangzhou, China; 8 Centre for Regenerative Medicine and Health, Hong Kong Institute of Science and Innovation, Chinese Academy of Sciences 5/F, 15 Science Park West Ave., Hong Kong Science Park, Park Shek Kok, New Territories, Hong Kong SAR, China; 9 Key Laboratory of Biological Targeting Diagnosis, Therapy and Rehabilitation of Guangdong Higher Education Institutes, The Fifth Affiliated Hospital of Guangzhou Medical University, Guangzhou, China; 10 Westlake Laboratory of Life Sciences and Biomedicine, Hangzhou, 310024 Zhejiang China

## Abstract

Nuclear condensates have been shown to regulate cell fate control, but its role in oncogenic transformation remains largely unknown. Here we show acquisition of oncogenic potential by nuclear condensate remodeling. The proto-oncogene SS18 and its oncogenic fusion SS18-SSX1 can both form condensates, but with drastically different properties and impact on 3D genome architecture. The oncogenic condensates, not wild type ones, readily exclude HDAC1 and 2 complexes, thus, allowing aberrant accumulation of H3K27ac on chromatin loci, leading to oncogenic expression of key target genes. These results provide the first case for condensate remodeling as a transforming event to generate oncogene and such condensates can be targeted for therapy.

**One sentence summary**: Expulsion of HDACs complexes leads to oncogenic transformation.

**Supplementary Information:**

The online version contains supplementary material available at 10.1186/s12943-024-02002-1.

## Introduction

Proto-oncogenes undergo mutagenic transformation to become oncogenic. For instance, RAS becomes oncogenic through V12 point mutation [1, 2]. Chromosomal translocation is a frequent mechanism that leads to dis-regulated kinases, transcription factors. Successful therapeutics have been developed based on these transformation events. For example, the Philadelphia chromosome that carries translocation between ch22 and 9, generating BCR-Abl which has been successfully targeted by Gleevac [3].

Biomolecular condensates formed by liquid–liquid phase separation (LLPS) widely exist in cells, which play an important role in many biological processes, including gene transcription regulation [4], innate immunity [5], and nerve conduction [6]. The underlying principle for these condensates formation is multivalent macromolecular interactions, like DNA/RNA molecules and proteins composed of multiple interaction domains and/or intrinsically disordered regions (IDRs) [7]. Recent studies have shown that the condensates of LLPS may play critical roles in the process of tumorigenesis [8–10]. Moreover, the recent work also has proposed that the alternative mechanism, like low-valency interactions with spatially clustered binding sites (ICBS), also contributions to condensate formation [11–13], which could be distinguished by half-FRAP [13]. However, whether the original condensates have been remodeled during this process, and the differences between the innate condensates and the corresponding onco-condensates are still unclear.

**Fig. 1:**
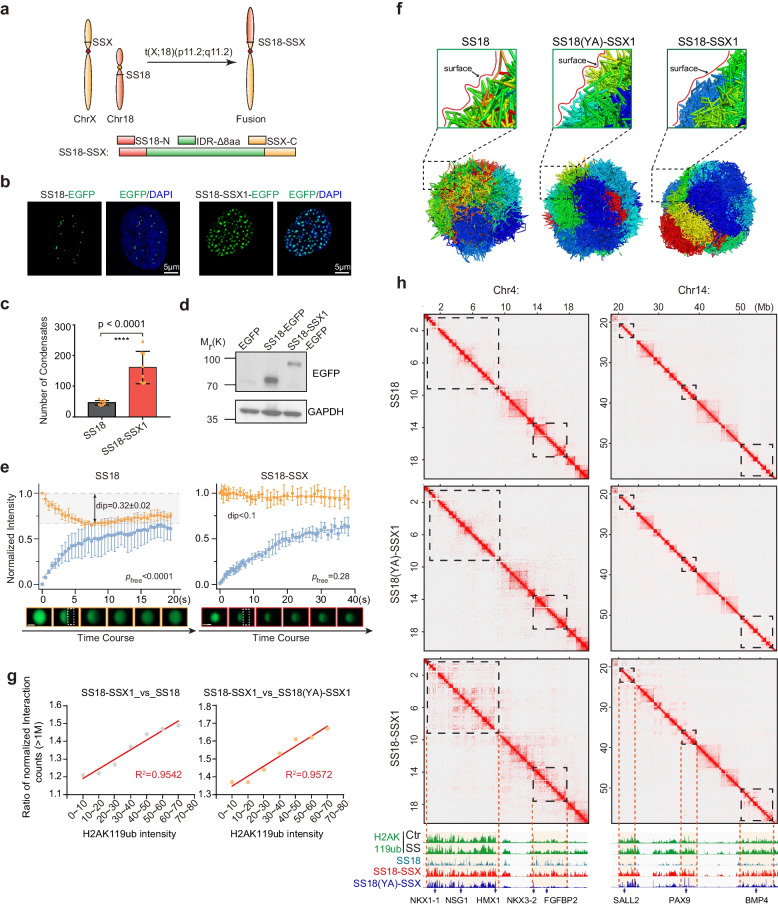
SS18-SSX condensates regulate long-range chromatin interaction. **a** Schematic illustration for SS18-SSX onco-fusion in synovial sarcoma. The C-terminal 8 amino acids of SS18 were replaced by 78 amino acids of SSX C terminus. **b** Representative images of SS18-EGFP and SS18-SSX1-EGFP fusion protein overexpressed via lentiviral infection in BJ fibroblasts. Scale bars, 5 μm. **c** Histogram for the number of condensates quantified from (**b**). Data are mean ± s.d., two-sided, unpaired t-test; *n* = 8 nuclei. **d** The expression of SS18-EGFP and SS18-SSX-EGFP were confirmed by western blotting. **e** Half-FRAP experiments performed after expression of SS18-EGFP or SS18-SSX1-EGFP protein in HEK293T cells. Top panels show half-FRAP curves, including the normalized intensity of the bleached half (blue) and the non-bleached half (yellow). The gray areas in the figure indicate the dip depth range of the unbleached half corresponding to the LLPS. Bottom panels show representative images at different time points before and after bleach. *p*-values based on a one-sided Student’s t-test against the dip depths obtained for free diffusion are indicated. Scale bars, 1 µm. *n* = 5 condensates. **f** Schematic illustration shows the 3D chromatin structures of the cells with expression of physiological SS18, condensates deficiency mutant SS18(YA)-SSX and pathological SS18-SSX, respectively. The construction process was described in the method of Hi-C data analysis. The upper panels were the magnified corresponding local surfaces. Each color represents a pair of homologous chromosomes. **g** Correlation analysis between the H2AK119ub intensity and the enhance ratio of long-range (more than 1 Mb) chromatin interaction comparing SS18-SSX with wildtype SS18 (the left) or condensate deficiency mutant SS18(YA)-SSX (the right). **h** The representative regions at chromosome 4 and 14 show the correlation of enhanced long-range chromatin interaction with H2AK119ub modification

Synovial sarcoma is a malignant tumor that occurs mainly in adolescents and young adults, accounting for 8%-10% soft-tissue malignancies [14, 15]. Almost all the synovial sarcomas carry chromosomal translocation, t (X,18; p11, q11), resulting in the fusion onco-protein SS18-SSX in which C terminus of SSX gene family (most SSX1, less SSX2 and rarely SSX4) in X chromosome replaced the 8 C-terminal amino acids of SS18 protein encoded by chromosome 18 [16, 17] (Fig. 1a). It has been shown that SS18-SSX fusion protein is both necessary and essential for synovial sarcomagenesis by recognizing H2AK119ub histone modification [18] and hijacking BAF and PRC1.1 complex [14, 17, 19, 20].

We have shown recently that SS18 protein regulates pluripotent-somatic transition (PST) through tyrosine-based phase separation mechanism [21]. Interestingly, the onco-fusion SS18-SSX protein also forms condensates [10]. It is not clear how SS18-SSX condensates acquire oncogenic activities. In this report, we show that the onco-condensates formed by SS18-SSX physically exclude HDACs and allow abnormal accumulation of H3K27ac at novel target sites.

## Method

### Cell culture

HEK293T cells (ATCC, CRL-1126) and SW982 cells are cultured in DMEM supplemented with 10% FBS, GlutaMAX and NEAA. BJ fibroblasts are cultured in DMEM/F12 supplemented with 10% FBS, GlutaMAX and NEAA. We used PRT4165 (TargetMol T3110) and A485 (MCE HY-107455) to block the H2AK119ub and H3K27ac modification, respectively. All the cell lines have been confirmed as mycoplasma free with Lonza LT07-318.

### qRT–PCR and RNA-seq

Total RNAs were prepared with TRIzol. For quantitative PCR, cDNAs were synthesized with ReverTra Ace (Toyobo) and oligo-dT (Takara), and then analysed by qPCR with ChamQ SYBR qPCR Master Mix (Vazyme). VAHTS mRNA-seq V3 Library Prep Kit for Illumina (NR611, Vazyme) was used for library constructions and sequencing done with NextSeq500 Mid output 150 cycles (FC-404–2001, Illumina) for RNA-seq.

### EdU cell proliferation assay

The proliferation of cells was detected using EdU cell proliferation assay according to the reagent instructions. About 3 × 10^4^ cells were seeded in 48-well plates and maintained for 24 h before the assay. A total of 200µL EdU (10 µM) reagent (Beyotime, C0078S) was added to each well and incubated for 6 h to label the cells. After two times wash with PBS, cells were fixed in a 4% paraformaldehyde solution (Sangon, E672002-0500) for 30 min, permeabilized with 0.3% Triton X-100 (Sigma-Aldrich, 9036–19-5) for 15 min, and then incubated with the click-reaction reagent for 30 min at room temperature in the dark environment. After the end of each of the above steps, wash 3 times with PBS containing 3% BSA for 3–5 min each time. In all, 1 × Hoechst33342 reagent was used to counterstain the nucleus. The result of staining was observed with a fluorescence microscope system ZEISS, and the data were collected by the ImageJ software.

### Immunoblotting

Cells were collected and lysed in lysis buffer supplemented with protease inhibitor cocktail (Roche) on ice for 15 min, and then cells were boiled in 100 °C for 10 min. After centrifugation, the cell supernatants were subjected to SDS–PAGE and incubated with corresponding primary antibody and secondary antibodies.

### Immunofluorescence

Cells growing on coverslips were washed 3 times with PBS, then fixed with 4% PFA for 30 min, and subsequently penetrated and blocked with 0.1% Triton X-100 and 3% BSA for 30 min at room temperature. Then, the cells were incubated with primary antibody for half hour. After 5 washes in PBS, one hour of incubation in secondary antibodies, cells were then incubated in DAPI (Sigma-Aldrich D9542) for 2 min. Then, the coverslips were mounted on the slides for observation on the confocal microscope (ZEISS, LSM 900).

### In situ Hi-C

Crosslink 1 million cells with 37% formaldehyde (sigma, F8775) to a final concentration of 1% and incubate for 10 min at room temperature. Add glycine (Yuanye, S20159) to a final concentration of 0.125 M, and terminate the formaldehyde crosslinking reaction at room temperature for 5 min. Cells were pelleted by spinning at 400 g for 5 min at 4 °C. The pellet was washed with cold PBS. Liquid nitrogen quick-freezes, placed at -80 degrees for reserve. Fixed cells were incubated in 500 μl of cold lysis buffer (10 mM Tris–HCl pH 8.0, 10 mM NaCl, 0.2% Igepal CA630 and protease inhibitors (MCE-HY-K0010)) on ice for 25 min. Cells were pelleted by spinning at 400 g for 5 min at 4 °C. The pellet was washed twice with 1xNEBuffer2. Cells were resuspended in 50 μl of 0.5%SDS and incubated for 8 min at 62 °C. Next, reactions were terminated with 145 μl of water and 25 μl of 10%Triton X-100 (Sigma, 93,443) at 37 °C for 15 min. Sample was digested overnight with 25 μl of 10X NEBuffer2 and 20 μl of MboI (NEB, R0147, 5U/µl) at 37 °C with rotation. Incubate the reactants at 62 °C for 20 min to inactivate MboI, then cold to room temperature. To complement the bases at the sticky end and label biotin at the DNA end, add 50 μl of the mixture (37.5 μl 0.4 mM biotin-14-dATP, 1.5 μl of 10mMdCTP, 1.5 μl of 10 mM dGTP, 1.5μlof 10 mM dTTP, and 8 μl of 5U/μl DNA polymerase I, large (Klenow) fragment(NEB M0210)) and incubating at 37 °C for 4 h. After that, ligation is performed by adding 120 μl of 10XNEB T4 DNA ligase buffer (NEB, B0202L), 100 μl of 10% Triton X-100, 637.8 μl of water, 1.2 μl of 10%BSA (Sigma-Aldrich SRE0098) and 5 μl of 400 U/μl T4 DNA ligase (NEB, M0202L) and incubating for 6 h at room temperature. De-crosslinking was performed by adding 50μL of 20 mg/mL proteinase K (NEB, P8107) and 120μL of 10%SDS incubate at 55 °C for 30 min. And then 130μL of 5 M NaCl was added and incubated at 68 °C overnight. Samples were cooled to room temperature and divided into three equal portions. Then, 800 μl of pure ethanol and 50 μl of 3 M sodium acetate pH5.2 were added to each tube which were subsequently incubated for 30 min at -80 °C. Spin the tubes at maximum speed for 15 min at 4 °C and wash twice with 70% ethanol. Resuspend the resulting pellet in 130 μl of 10 mM Tris–HCl, pH8.0 and incubate at 37 °C for 15 min. DNA was sheared using an ME220 Covaris Focused-ultrasonicator to a fragment size of 300–500 bp. Sheared DNA was size selected using VAHTS® DNA Clean Beads N411. Elution was done with hot (65 °C) 70 μl EB buffer. Next, the sample is transferred to a DNA LoBind tube (Eppendorf, 30108418) for related experimental manipulation. Take 150ul of T1 beads (MyOne Streptavin T1 Beads, Invitrogen, 65601) into a DNA LoBind Tubes and add 500ul 1 × TWB buffer (5 mM Tris–HCl (pH 7.5); 0.5 mM EDTA; 1 M NaCl; 0.05% Tween 20) wash twice. Resuspend the T1 beads with 70ul 2 × Binding Buffer (10 mM Tris–HCl (pH 7.5), 1 mM EDTA, 2 M NaCl) and combined with 70 μl Hi-C DNA from the previous step. The mixture was incubated at room temperature for 15 min. After which the magnet adsorbed to remove the supernatant; Wash twice with 200ul 1 × TWB buffer and once with 200ul EB buffer. Finally, resuspend the beads with 50ul of water and transfer to a 200ul PCR tube. Library construction using the VAHTS Universal DNA Library Prep Kit (Vazyme ND607) and VAHTS Multiplex Oligos Set 4/5 kits (Vazyme N321/N322) according to manufacturer’s instructions.

### Hi-C data analysis

The 20 kb chromatin bins (hg38) interaction matrix was obtained by HiC-Pro (Version 3.1.0) with GATCGATC ligation site. The duplicate reads and the invalid interactions were removed by HiC-Pro. Juicer tools software (Version 1.22.01) was used for visualization. The interaction count between different bins was normalized by the sample’s total number of counts. The signal of H2AK119ub modification was obtained from CUT&Tag data and the bins with the signal more than 40 were considered as the H2AK119ub modified regions. For the 3D Structure Reconstruction, Hi-C reads were remapped by BWA-MEM (Version 0.7.17) to the reference human genome (hg38) with default parameters. Then contact pairs were extracted using hickit (Version r291). We next reconstructed the 3D structure using hickit with the parameters “-s1 -r1m -c1 -r10m -c2 -b4m -b1m -b200k -D5 -b50k -D5 -b20k -D5 -b10k”. The structures were visualized using PyMOL (Version 2.5.5).

### CUT&Tag and data analysis

The CUT&Tag experiments of SS18, SS18-SSX, H2AK119ub and H3K27ac were constructed with NovoNGS CUT&Tag 2.0 High-Sensitivity Kit for Illumina (novoprotein N259-YH01) according to manufacturer’s instructions. Reads form ChIP-seq experiments were mapped to the human genome (hg38) using Bowtie2 (–very-sensitive), and only those reads that mapped once were retained for further analysis. Peaks were called using MACS2 software with the default parameters.

### Plate colony-formation assay

The SW982 cells stably expressed empty vector, wild-type SS18-SSX1 and its mutants were seeded at 1000 cells per well in six-well plates. Cells were cultured supplemented with 15% FBS. Cells were cultured at 37 °C for 10 days. The culture medium was changed every 3 days. After 10 days, cells were fixed in 4% paraformaldehyde (Sangon, E672002-0500) for 20 min at room temperature and then stained with crystal violet (Beyotime, C0121-100 ml) for 10 min at room temperature. Cells were washed with water three times and air dried. Finally, the bottom of the 6-well plate was scanned with a scanner (HuiPu,HPD49FD8) and the number of cell clones was statistically analyzed.

### Cell-derived xenograft study

In brief, 6-week-old female NCG mice (Gempharmatech, T001475) were subcutaneously implanted with 5.0 × 10^6^ SW 982 cells overexpressing SS18-SSX1-EGFP in 50% Matrigel (Corning, 354234)/Leibovitz's L-15 medium (Gibco, 11415064). Tumor volumes were measured by caliper and mice were weighed every three days. Mice were randomized into two groups (*n* = 4) once tumor volume reached approximately 100 mm^3^ (V = L × W^2^/2): vehicle (10% DMSO (Sigma-Aldrich, D2650) + 40% PEG300 (MedChemExpress, HY-Y0873) + 5% Tween 80 (MedChemExpress, HY-Y1891) + 45% saline) or A-485 (100 mg/kg/dose, 2 dose/day) by intraperitoneal injection. Mice were euthanized according to institutional guidelines when tumor volumes equaled to 15% of body weights or if mice became moribund, with tumor being extracted and divided for IHC.

### Statistical information

Data are presented as mean ± s.d. as indicated in the figure legends. Unpaired two-tailed student t-test, The *P*-Value was calculated with the Prism 6 software. A *P*-Value < 0.05 was considered as statistically, *p < 0.05, **p < 0.01, ***p < 0.001, ****P < 0.0001. No statistical method was used to predetermine sample size. The experiments were not randomized. The investigators were not blinded to allocation during experiment and outcome assessment.

## Results

### SS18-SSX forms novel condensates and re-organizes the 3D genome

In synovial sarcoma samples, there is a chromosome translocation that results in the replacement of C-terminal 8 amino acids of wild type SS18 by 78 amino acids of SSX genes (SSX1, SSX2 or SSX4) to form onco-protein SS18-SSX (Fig. 1a). To better understand this Onco-transformation event, we show that SS18-SSX1 can induce extensive activation of embryonic developmental genes (Supplementary Fig. 1a, b), like PAX6, NKX3-2 and SOX2, and the down regulation of genes are involved in extracellular matrix/structure organization (Supplementary Fig. 1c). To understand the molecular basis of transforming SS18 into oncogenic SS18-SSX, we analyzed their condensates and show that SS18-SSX is at least 300% more efficient than SS18 (Fig. 1b, c), with less protein expressed on western blot (Fig. 1d). We next performed half-FRAP assay [13] to probe the nature of SS18/SS18-SSX condensates and show that the unbleached half condensates exhibit distinct molecular dynamics (Fig. 1e), suggesting that the mechanism of SS18-SSX condensate formation might be closer to low-valency interactions with spatially clustered binding sites (ICBS) than to the liquid–liquid phase separation (LLPS). It has been reported that SS18-SSX could recognize H2AK119ub histone modification [18], which can provide the scaffold for low-valency interactions. To test it, we mutated all the acidic amino acids which has been reported to recognize H2AK119ub [18], by alanine in the C terminus of SSX1. We show that the resulting mutant fails to form condensates (Supplementary Fig. 2a-c), suggesting that acid residue mediated low-valency interaction is responsible for the emerging property of SS18-SSX.

We then tested the role of tyrosine residues in SS18 IDR previously shown to be critical for condensate formation of SS18 protein [21], and show that once the tyrosine enriched in the SS18-IDR mutated totally, SS18-SSX also fails to form condensates in comparison with other enriched amino acids Gln, Gly and Pro (Supplementary Fig. 3a, b). Furthermore, although SS18(YA)-SSX mutant loses the capability to form condensates, it remains competent in recognizing and binding to chromatin regions with H2AK119ub histone modification (Supplementary Fig. 3c, d). However, SS18(YA)-SSX mutant fails to activate the downstream genes (Supplementary Fig. 3e) and loses tumorigenicity based on EdU cell proliferation assay (Supplementary Fig. 3f, g), colony formation assay (Supplementary Fig. 3h, i) and tumor-bearing mouse models (Supplementary Fig. 3j-l), suggesting that the tyrosine-based condensation also plays a critical role in its tumorigenic potential.

Given the more pronounced condensate formation phenomenon of pathological SS18-SSX condensates than wild type SS18 condensates (Fig. 1b, c), we hypothesize that it may be able to transform the 3D genome architecture. To test this, we performed Hi-C experiments to probe the effect of SS18-SSX onco-fusion on 3D chromatin structure. Compared to wild type SS18 and condensate-deficient SS18(YA)-SSX, SS18-SSX nuclei appear to be smoother, consistent with a more ordered structure (Fig. 1f), characteristic of enhanced long-range chromatin interaction (Supplementary Fig. 4a, b). Consistently, this enhancement occurs more frequently in regions with H2AK119ub modification than those without (Supplementary Fig. 4c-f, Fig. 1h). Additionally, compared with wild type SS18 or condensate-deficient mutant SS18(YA)-SSX, enhancement of long-range (more than 1 M) chromatin interaction by SS18-SSX is positively correlated with the intensity of H2AK119ub modification (Fig. 1g). Similar structural changes could also be observed when tested in IMR-90 cells (Supplementary Fig. 4g, h). These results suggest that SS18-SSX possess a unique ability to promote long-range chromatin interactions and a more compact architecture by at least enriching H2AK119ub modified chromatin.

### SS18-SSX condensates exclude HDACs

**Fig. 2:**
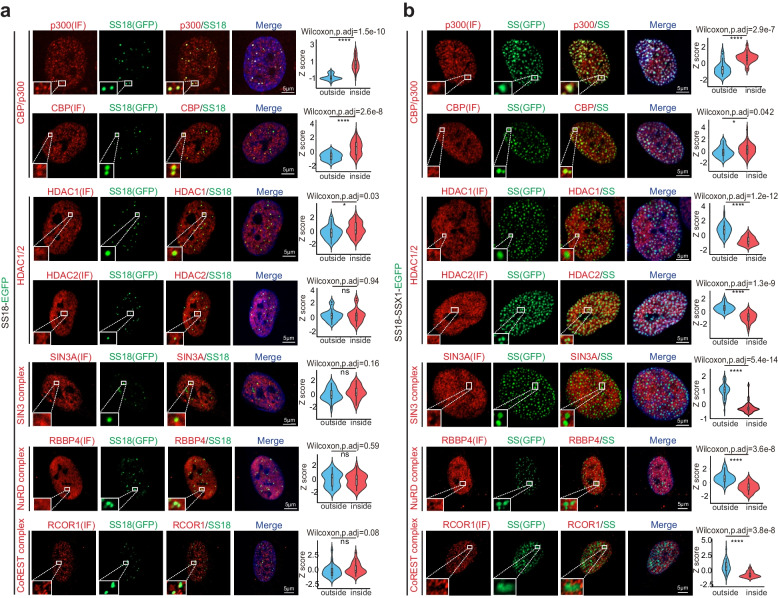
SS18-SSX1 excludes HDAC1/2 complexes from condensates. Representative immunofluorescent images of endogenous p300/CBP, HDAC1/2 or HDAC1/2 associated transcription repressive complexes SIN3/NuRD/CoREST and lentiviral expression of SS18-EGFP (**a**) or SS18-SSX-EGFP (**b**) on the left panels. Scale bars, 5 μm. The violin plots on the right panels show the quantitative analysis of co-localization. Outside and inside groups indicate the distribution of random pixels’ fluorescence intensity normalized by Z score from outside and inside of SS18 (**a**) or SS18-SSX (**b**) condensates. Two-sided Wilcoxon test adjusted for multiple comparisons. *n* = 30 pixels, from 3 nuclei. *p < 0.05, ****p < 0.0001. ns, not significant. SS, SS18-SSX1

The new properties emerged from SS18-SSX fusion suggest that it may elicit new function inside nuclei. To test this hypothesis, we analyzed several candidate biomacromolecular complexes like BRG1/BAF we previously reported [21]. For example, we show that SS18 innate or native condensate could enrich acetyltransferase CBP/p300 while allowing free access of HDAC1/2 (the most abundant HDACs in BJ cells) and corresponding repressor complexes containing HDAC1/2, like SIN3 complex (SIN3A), NuRD complex (RBBP4) and CoREST (RCOR1), into the interior of the condensates (Fig. 2a). Interestingly, while SS18-SSX onco-condensates remain competent in enriching CBP/p300 (Fig. 2b) and BRG1 (Supplementary Fig. 5a), it readily excludes HDAC1/2 and corresponding repressor complexes (Fig. 2b), which could also be observed by HDAC1/2 CUT&Tag experiment (Supplementary Fig. 5b, c). We also show that the exclusion behavior of HDACs also occurs in endogenous SS18-SSX condensates engineered by gene editing in human embryonic stem cells (hESCs) (Supplementary Fig. 6a-c), synovial sarcoma cell line HS-SY-II (Supplementary Fig. 6i) and in primary tumor cells from synovial sarcoma patients (Supplementary Fig. 6j). Interestingly, the knock-in of C-terminal SSX at the SS18 locus led to the activation of genes for neural differentiation (Supplementary Fig. 6d, e) and neural rosette-like structures in hESCs (Supplementary Fig. 6g, h) without intervening pluripotent genes expression (Supplementary Fig. 6f). These results suggest that SS18-SSX acquire the ability to exclude repressors through its oncogenic translocation.

### Forced re-entry of HDAC1 into SS18-SSX condensates neutralizes its oncogenic potential

**Fig. 3:**
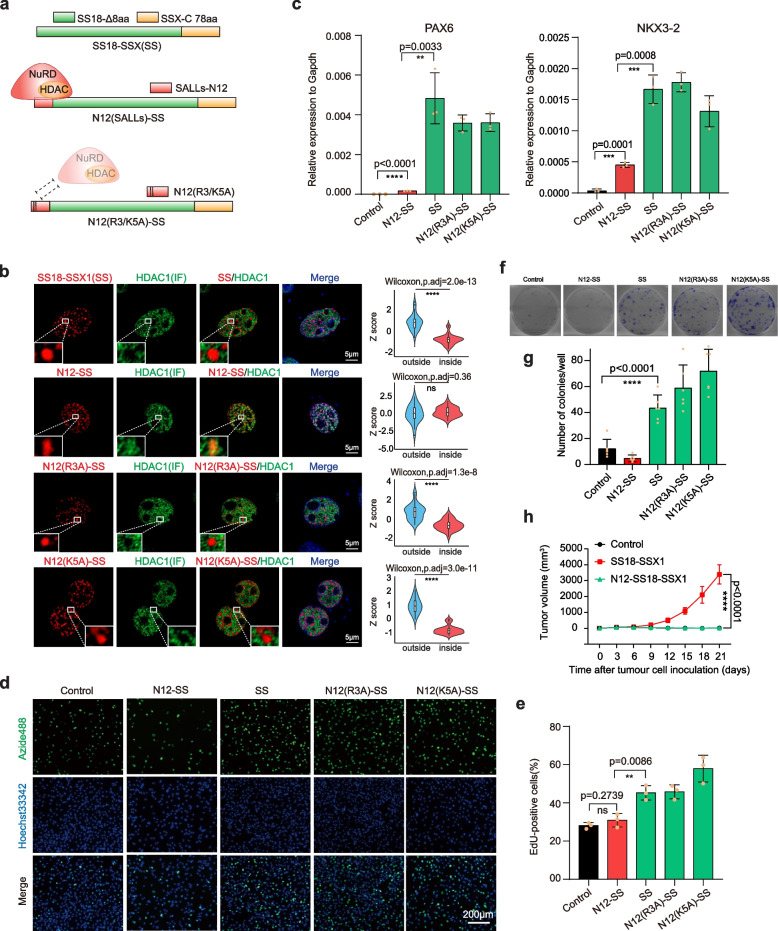
Interference HDACs complexes exclusion alleviates oncogenicity of SS18-SSX condensates. **a** Schematic illustration shows the SS18-SSX fused transcription factor SALLs derived N12 domain in the N terminus could recruit HDAC1/2 involved NuRD complex. N12 with R3A or K5A mutation loses that capability. SS, SS18-SSX. **b** Left panels are representative immunofluorescent images of endogenous HDAC1 in the BJ cells with lentiviral expression of SS18-SSX, N12 fused SS18-SSX and N12 with R3A or K5A mutation fused SS18-SSX, respectively. Scale bars, 5 μm. The violin plots on the right panels show the quantitative analysis of co-localization. Outside and inside groups indicate the distribution of random pixels’ fluorescence intensity normalized by Z score from outside and inside of SS18-SSX, N12 fused SS18-SSX and N12 with R3A or K5A mutation fused SS18-SSX condensates, respectively. Two-sided Wilcoxon test adjusted for multiple comparisons. *n* = 30 pixels, from 3 nuclei. ****p < 0.0001. ns, not significant. SS, SS18-SSX1. **c** The expression of representative downstream genes of SS18-SSX in BJ fibroblasts with lentiviral expression of EGFP, SS18-SSX, N12 fused SS18-SSX and N12 with R3A or K5A mutation fused SS18-SSX, respectively. Data are mean ± s.d., two-sided, unpaired t-test, **p < 0.01, ***p < 0.001, ****p < 0.0001. *n* = 3 independent experiments. SS, SS18-SSX1. **d** Representative image of EdU assay in synovial sarcoma cell line SW982 with lentiviral expression of (**b**). Scale bars, 200 μm. SS, SS18-SSX1. **e** Histogram shows the ratio of EdU positive cells of (**d**). Data are mean ± s.d., two-sided, unpaired t-test, **p < 0.01, ns, not significant. *n* = 3 independent experiments. SS, SS18-SSX1. **f** Representative images of colony formation assay in synovial sarcoma cell line SW982 overexpressing mCherry, SS18-SSX, N12 fused SS18-SSX and N12 with R3A or K5A mutation fused SS18-SSX, respectively. One well of a 6-well plate was seeded with 1,000 cells and cultured for 10 days. Colonies were stained with 0.1% crystal violet. SS, SS18-SSX1. **g** Histogram shows the number of colonies in one well from (**f**). Data are mean ± s.d., two-sided, unpaired t-test, ****p < 0.0001, *n* = 6, from 3 independent experiments. SS, SS18-SSX1. **h** Tumor growth curve of SW982 cell overexpressing mCherry, SS18-SSX and N12 fused SS18-SSX xenografts established in NCG mice for 21 days. Data are mean ± s.d., two-sided, unpaired t test of *n* = 4 mice per group from two biological replicates, ****p < 0.0001

The exclusion of HDACS further intrigues us to probe the underlying mechanisms. First, we wish to test if endogenous HDACs can be forced to return to the condensates. To this end, we took advantage of the well-known N12(Sall4)-NuRD interaction module [22, 23] and fuse it or its variants to SS18-SSX (SS) and show that N12, not its variant N12(R3A) nor N12(K5A), can pull endogenous HDAC1 into the condensates (Fig. 3a, b), presumably as part of NuRD complex [22, 23]. Restoration of HDAC complexes in the condensates severely dampens the activation of SS18-SSX downstream genes such as PAX6 and NKX3-2 (Fig. 3c) and almost abolishes the tumorigenicity of SS18-SSX tested by EdU cell proliferation assay (Fig. 3d, e), colony formation assay (Fig. 3f, g) and tumor-bearing mouse models (Fig. 3h, Supplementary Fig. 7a, b). These results suggest that the active exclusion of HDAC repressor complexes by SS18-SSX onco-condensates may play a critical role in synovial sarcomagenesis.

Re-entry of HDAC1 to SS18-SSX condensates via N12 further encouraged us to probe the exclusion mechanism. To this end, we focused on HDAC1, which has N-terminal histone deacetylase and C-terminal disordered region (Supplementary Fig. 8a). By deletion analysis, we show that its C-terminal disordered region is both required and sufficient for exclusion by SS18-SSX condensates (Supplementary Fig. 8b). We then analyzed the amino acid distribution of HDAC1 and found that its C terminus is enriched with acidic amino acids (Glu and Asp), basic amino acids (Lys) and serine (Supplementary Fig. 9a). We performed mutation analysis and show that mutating the charged residues render HDAC1 accessible again into SS18-SSX1 condensates, especially the one on acid residues, i.e., HDAC1(A) (Supplementary Fig. 9b). These results provide preliminary insight into the inclusion and exclusion of protein complexes into condensate.

### SS18-SSX condensates accumulate H3K27ac that can be targeted pharmacologically

**Fig. 4:**
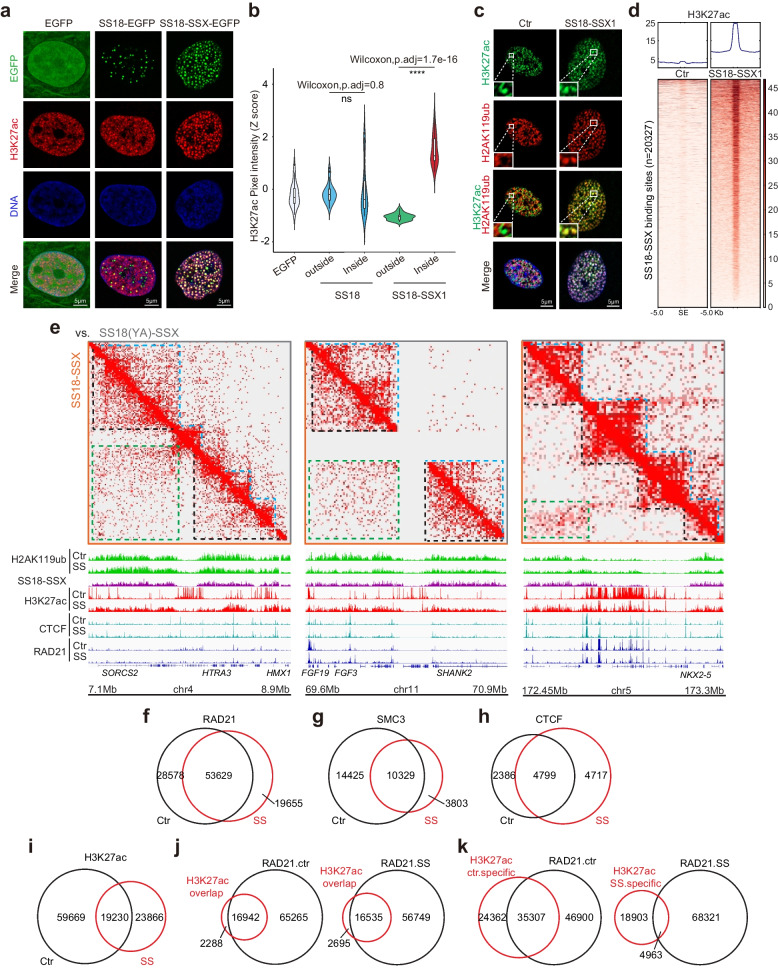
Onco-condensate remodels H3K27ac. **a** Representative image of immunofluorescence for H3K27ac in the BJ fibroblasts with lentiviral expression of SS18-EGFP or SS18-SSX1-EGFP. Scale bars, 5 μm. **b** Violin plot shows the pixel intensity distribution of immunofluorescence for H3K27ac in (**a**). Outside and inside groups indicate the distribution of random pixels’ fluorescence intensity of H3K27ac normalized by Z score from outside and inside of SS18 or SS18-SSX1 condensates, respectively. Two-sided Wilcoxon test adjusted for multiple comparisons. *n* = 30 pixels, from 3 nuclei. ****p < 0.0001. **c** Representative image of immunofluorescence for H3K27ac and H2AK119ub in the BJ fibroblasts with lentiviral expression of SS18-SSX1. Scale bars, 5 μm. **d** Heatmap shows the intensity changes of H3K27ac occupancy at SS18-SSX1 binding sites upon expression of the onco-fusion protein. **e** Jointly analysis of Hi-C, CUT&Tag of histone modification, SS18-SSX1 and cohesin-CTCF at three representative loci in BJ fibroblasts with the expression of SS18-SSX or condensate-deficient SS18(YA)-SSX as control. The green boxes indicate the newly formed long-range interaction. The blue and black dotted lines indicate the intrinsic chromatin domains. **f** Venn plot shows the intersection of cohesin component RAD21 binding sites in BJ cells with the overexpression of SS18-SSX1 and SS18(YA)-SSX as control. SS, SS18-SSX1. **g** Venn plot shows the intersection of cohesin component SMC3 binding sites in BJ cells with the overexpression of SS18-SSX1 and SS18(YA)-SSX as control. SS, SS18-SSX1. **h** Venn plot shows the intersection of CTCF binding sites in BJ cells with the overexpression of SS18-SSX1 and SS18(YA)-SSX as control. SS, SS18-SSX1. **i** H3K27ac histone modification sites were divided into three groups by Venn plot, i.e., control specific sites, SS18-SSX1 specific sites and the overlapped sites. **j** The colocalization analysis between the overlapped H3K27ac sites in (**i**) with cohesin component RAD21 binding sites in BJ cells with the overexpression of SS18-SSX1 and SS18(YA)-SSX as control, respectively. SS, SS18-SSX1. **k** The colocalization analysis between the specific H3K27ac sites in (**i**) with cohesin component RAD21 binding sites in BJ cells with the overexpression of SS18-SSX1 and SS18(YA)-SSX as control, respectively. SS, SS18-SSX1

One of the consequences of HDAC exclusion may be the accumulation of acetylated histones. To test this hypothesis, we focused on H3K27ac and show that H3K27ac distribution is dramatically different between SS18 to SS18-SSX (Fig. 4a, b), with significant enrichments in the onco-condensates, which has not been observed from the other common active epigenetic modifications, such as H3K9ac, H3K4me3, and H3K36me2/3 (Supplementary Fig. 10a), and indeed, the interference of HDACs exclusion via N12 would significantly reduce the abnormal deposition of H3K27ac and the downstream gene expression (Supplementary Fig. 10b). Dramatically, H3K27ac and H2AK119ub have been transformed from mutually exclusive to inclusive as a result of SS18-SSX1 expression (Fig. 4c). Consequently, SS18-SSX1 is able to endow de novo H3K27ac in the entire SS18-SSX binding sites as shown by CUT&Tag experiments (Fig. 4d), although the intensity of global H3K27ac modification has been attenuated, especially at the intrinsic SS18 binding sites (Supplementary Fig. 11a-c). Additionally, the H3K27ac sites are more localized at 5’UTR and distal intergenic regions upon SS18-SSX expression (Supplementary Fig. 11d).

The dramatic co-localization of H3K27ac, H2AK119ub (a gene silencing marker by PRC1 [18]), and SS18-SSX, but not SS18, highlights extensive remodeling of chromatin when HDACs are excluded. We found the occupancy of H2AK119ub modification and SUZ12/PRC2 decreased and BMI1/PRC1 increased at the sites with dynamic changes of H3K27ac/me3, except for just a few genes, like NKX3-2 (Supplementary Fig. 11e-g).

We next analyzed the histone modification and Hi-C data combined with cohesin-CTCF, we show that the newly formed long-range interaction between H2AK119ub modified chromatin upon SS18-SSX expression could cross the boundary of the original chromatin domain established by cohesin-CTCF and endows aberrant H3K27ac deposition at these sites that were supposed to be silent (Fig. 4e). In comparison to condensate-deficient mutant SS18(YA)-SSX control, the expression of SS18-SSX in BJ cells attenuates the occupancy of cohesin but enhances the CTCF binding moderately (Supplementary Fig. 12a). Additionally, cohesin-CTCF shares most of the binding sites in the control and SS18-SSX overexpressed BJ cells (Fig. 4f-h). Intriguingly, when the total H3K27ac sites are divided into three groups, i.e., control specific sites, SS18-SSX1 specific sites and the overlapped sites (Fig. 4i, Supplementary Fig. 12b), we show that both the control specific and the overlapped sites have a large overlap with cohesin-CTCF binding sites, but the newly formed SS18-SSX1 specific sites are independent with cohesin-CTCF (Fig. 4j, k, Supplementary Fig. 12c-f). These data indicate that the oncogenic condensates formed by SS18-SSX may have created special microenvironment to enrich chromatin with H2AK119ub and CBP/p300, and simultaneously exclude HDAC1/2 complexes, which makes it favorable to deposit H3K27ac at these regions and activate the expression of downstream genes. Indeed, downstream genes activated by SS18-SSX indeed contain a stronger H2AK119ub modification (Supplementary Fig. 12g).

**Fig. 5:**
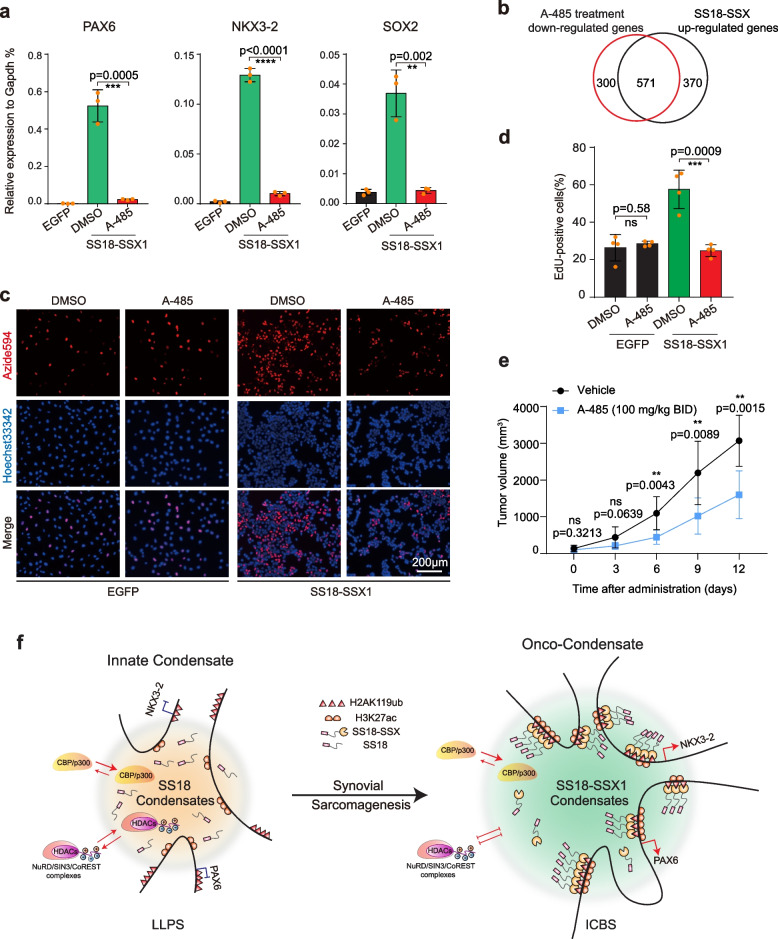
H3K27ac deposition can be targeted pharmacologically. **a** The expression of representative genes in BJ fibroblasts expressing control or SS18-SSX1 treated with DMSO or 1 μM A485 inhibitor. Data are mean ± s.d., two-sided, unpaired t-test; *n* = 3 independent experiments. **p < 0.01, ***p < 0.001, ****p < 0.0001. **b** Venn plots showing the intersection between upregulated genes and A485 treatment downregulated genes in BJ fibroblasts expressing SS18-SSX1. **c** Representative image of EdU assay in synovial sarcoma cell line SW982 with lentiviral expression of EGFP or SS18-SSX1 treated by DMSO or 1 μM A485. Scale bars, 200 μm. **d** Histogram shows the ratio of EdU positive cells in (**c**). Data are mean ± s.d., two-sided, unpaired t-test, *n* = 4, from 2 independent experiments. **e** Tumor growth curve of SW982 cell overexpressing SS18-SSX1-EGFP xenografts established in NCG mice. Mice were treated intraperitoneally with vehicle control or A-485 at 100 mg/kg/dose, twice daily for 12 days (BID × 12). Data are mean ± s.d., two-sided, unpaired t test of *n* = 7 mice per group from two biological replicates, **p < 0.01. **f** A Model for condensate remodeling as a driver for synovial sarcoma. SS18 WT protein forms innate phase separation for its normal physiological function (left panel). In contrast, SS18-SSX1 has enhanced condensates formation tendency mediated by ICBS mechanism via H2AK119ub histone modification and remodels 3D genome structure (right panel). The enhanced onco-fusion condensates remodel H3K27ac by excluding HDACs complexes, which resulting in aberrant activation of embryonic developmental genes

The imbalance of H3K27ac modification created by HDAC exclusion may be corrected by CBP/p300 inhibition. We tested this hypothesis with the compound A-485, a highly selective and drug-like CBP/p300 catalytic inhibitor [24], to decrease the over-deposited H3K27ac modification in SS18-SSX condensates (Supplementary Fig. 13a). We show that A-485 dramatically impede the activation of SS18-SSX downstream genes (Fig. 5a). A-485 treatment also reduces the expression of over 60% genes up-regulated by SS18-SSX1 (Fig. 5b) but has minimal impact on down-regulated and none-regulated genes (Supplementary Fig. 13b). Consistently, A-485 blocks tumorigenicity of onco-condensate validated by EdU cell proliferation assay (Fig. 5c, d) and tumor-bearing mouse models constructed by SW982 with overexpression of SS18-SSX (Fig. 5e, Supplementary Fig. 13c, d) and synovial sarcoma cell line HS-SY-II (Supplementary Fig. 13e-g), which suggested that targeting H3K27ac histone modification may be efficacious for treating synovial sarcoma carrying SS18-SSX.

## Discussion

We show here that chromosome translocation between 18 and X leads to a fusion protein with totally different properties, leading to oncogenic transformation by 3D genomic structure modeling and excluding HDACs, thus, creating an imbalance between H3K27ac and H2AK119ub genome wide. And this imbalance favors the expression of previously silenced genes that results in cell fate transition towards onco-fate (Fig. 5). We further show that this can be targeted pharmacologically with CBP/p300 inhibitors.

This work illuminates how physical properties of condensates can be radically remodeled to acquire new function, such as the exclusion of inhibitory regulators such as HDACs as an oncogenic event. Further studies are required to unravel the biophysical mechanisms associated with this transformation and reveal basic laws of condensate formation and remodeling inside the nuclei.

## Conclusion

Our study explored the relationship between condensates formation and tumorigenesis, revealed the mechanism of oncogenic fusion protein SS18-SSX mediating carcinogenesis through remodeling 3D genome structure and excluding HDACs complexes, and found that H3K27ac enriched on SS18-SSX1 condensates may be a new target for the treatment of synovial sarcoma.

### Supplementary Information

**Supplementary Material 1.**

**Supplementary Material 2.**

## Data Availability

The RNA-Seq, ChIP-seq and Hi-C data have been deposited in the Gene Expression Omnibus database under the accession code GSE211456, GSE263429 and GSE263435.

## Abbreviations

LLPS: Liquid-liquid Phase Separation
IDR: Intrinsically Disordered Region
ICBS: Interactions with spatially clustered binding sites
PST: Pluripotent-somatic Transition
qRT-PCR: Quantitative Real-time PCR
RNA-seq: RNA Sequencing
Hi-C: High-throughput Chromosome Conformation Capture
CUT&Tag: Cleavage Under Target &Tagmentation
hESC: Human Embryonic Stem Cells
