# Supplementary material for: Exclusion of HDAC1/2 complexes by oncogenic nuclear condensates
Source: Mol Cancer. 2024 Apr 27;23:85. doi: 10.1186/s12943-024-02002-1 (PMC11055323; doi:10.1186/s12943-024-02002-1)
Supplement: Supplementary file 1 — Supplementary Material 1. [file 12943_2024_2002_MOESM1_ESM.pdf]

Figure S1

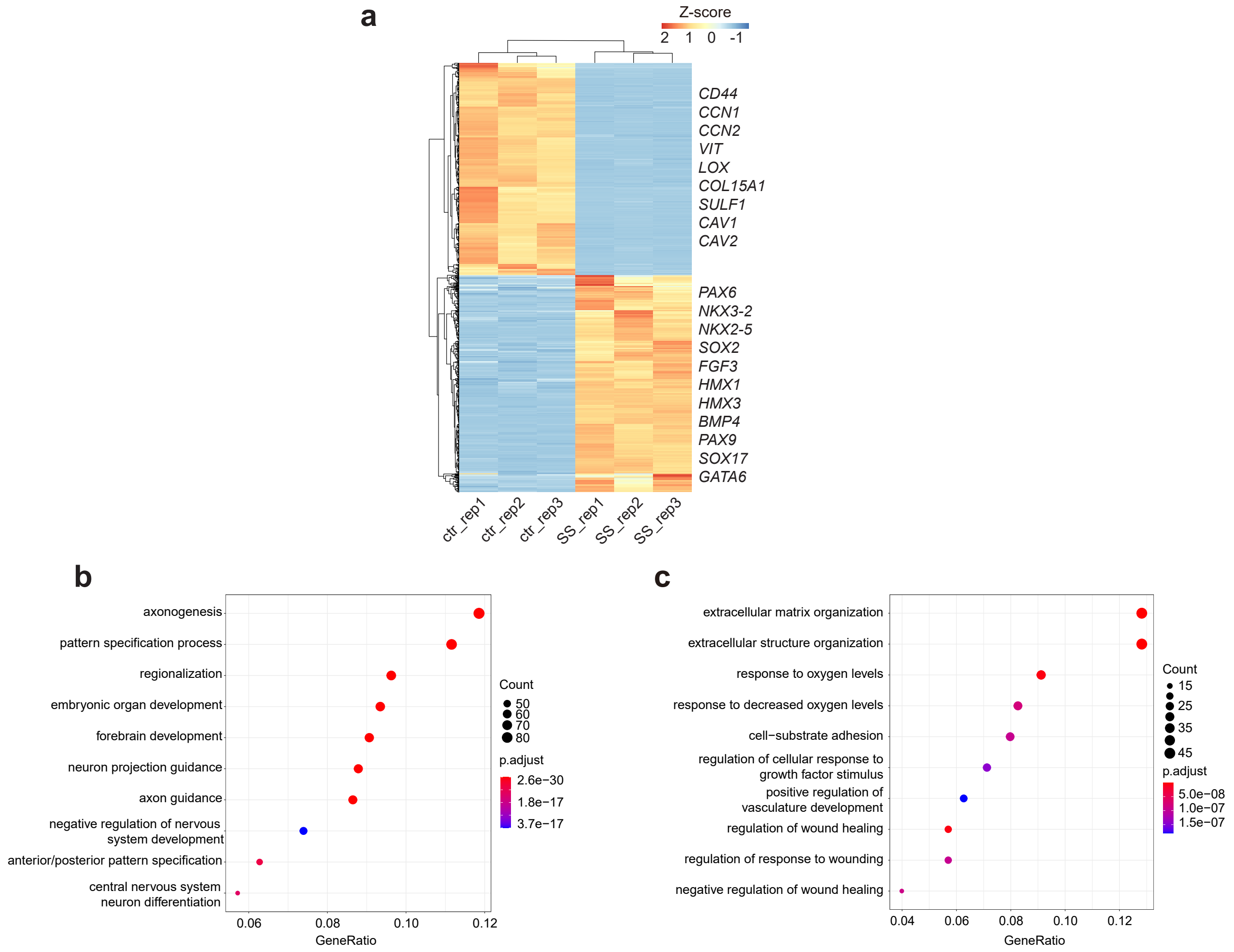
a
Z-score
2 1 0 -1
CD44
CCN1
CCN2
VIT
LOX
COL15A1
SULF1
CAV1
CAV2
PAX6
NKX3-2
NKX2-5
SOX2
FGF3
HMX1
HMX3
BMP4
PAX9
SOX17
GATA6
ctr_rep1
ctr_rep2
ctr_rep3
SS_rep1
SS_rep2
SS_rep3
b
axonogenesis
pattern specification process
regionalization
embryonic organ development
forebrain development
neuron projection guidance
axon guidance
negative regulation of nervous system development
anterior/posterior pattern specification
central nervous system neuron differentiation
0.06
0.08
0.10
0.12
GeneRatio
Count
50
60
70
80
p.adjust
2.6e−30
1.8e−17
3.7e−17
c
extracellular matrix organization
extracellular structure organization
response to oxygen levels
response to decreased oxygen levels
cell−substrate adhesion
regulation of cellular response to growth factor stimulus
positive regulation of vasculature development
regulation of wound healing
regulation of response to wounding
negative regulation of wound healing
0.04
0.06
0.08
0.10
0.12
GeneRatio
Count
15
25
35
45
p.adjust
5.0e−08
1.0e−07
1.5e−07

Supplementary Figure legends

Supplementary Fig. 1

Differential gene expression profile upon SS18-SSX1 aberrant expression.

a. Heat map showing the differential expressing genes in BJ fibroblasts expressing EGFP as control or SS18-SSX1. Upregulated 941 genes and downregulated 1033 genes.
b. Gene ontology analysis showing the expression pattern of upregulated genes after BJ fibroblasts expressing SS18-SSX1.
c. Gene ontology analysis showing the expression pattern of downregulated genes after BJ fibroblasts expressing SS18-SSX1.

Figure S2

a

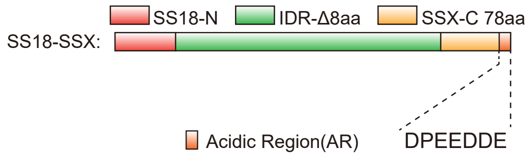

b

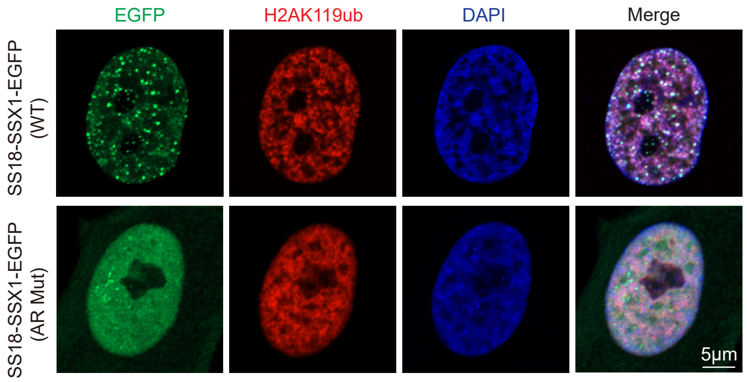

c

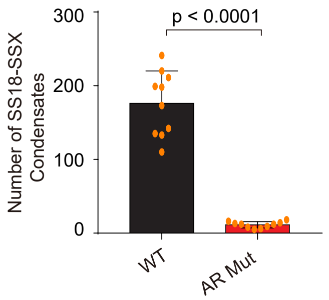

Supplementary Fig. 2

Formation of the SS18-SSX1 condensates depends on H2AK119ub recognition.

a. Schematic illustration for SS18-SSX onco-fusion in synovial sarcoma. The 7 amino acid sequence of the C-SSX acidic region (AR) is marked.

b. Representative images of immunofluorescence of H2AK119ub in BJ fibroblasts expressing wild-type or AR-mutant SS18-SSX1-EGFP protein. Scale bar, 5 μm. All the 6 acidic amino acids in AR were mutated by alanine.

c. Histogram for the number of condensates quantified from (b). Data are mean±s.d., two-sided, unpaired t-test; ****$p < 0.0001$. $n=8$ nuclei.

Figure S3

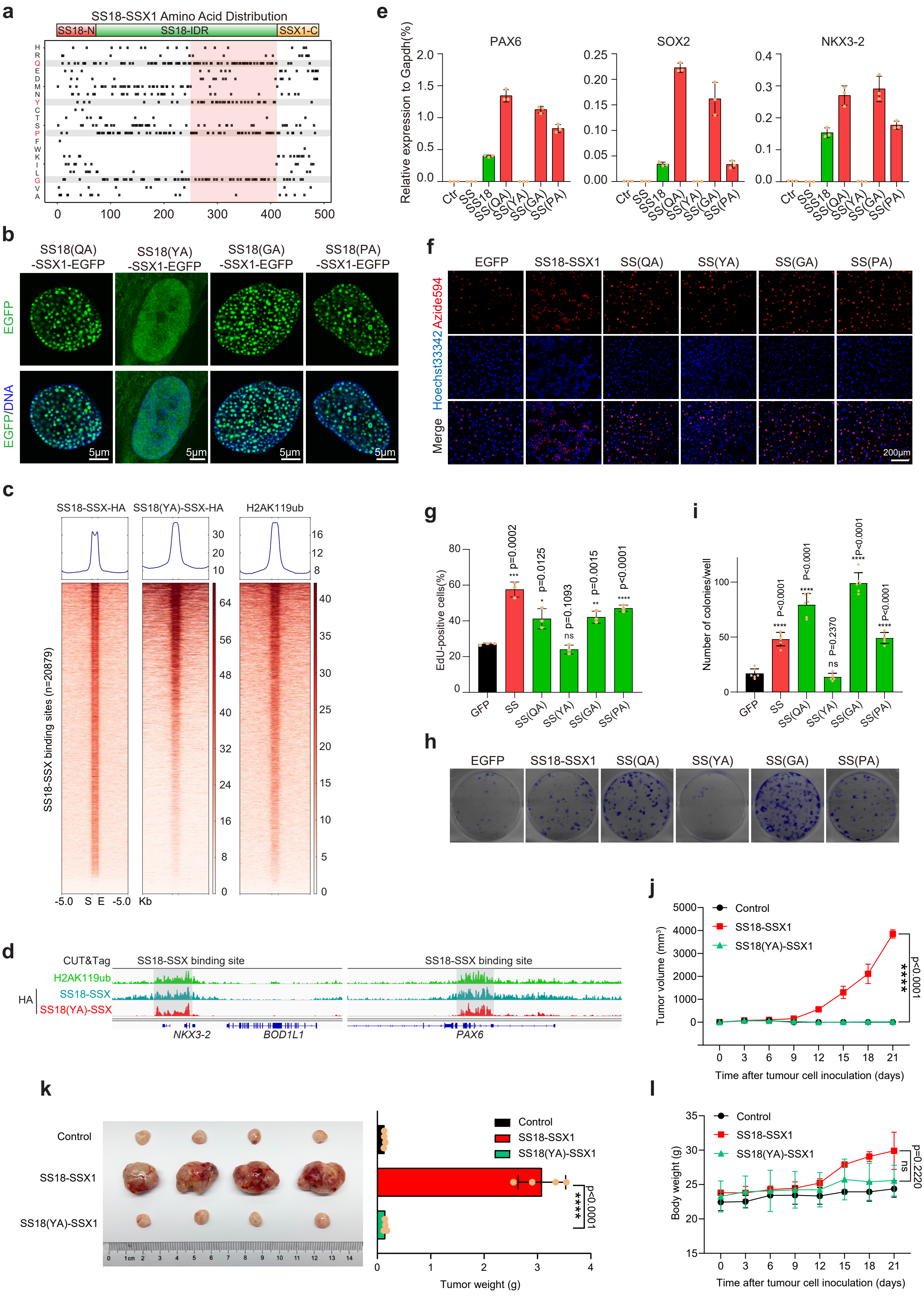

a
SS18-SSX1 Amino Acid Distribution
SS18-N
SS18-IDR
SSX1-C
H R Q E D M N Y C T S P F W K I L G V A
0 100 200 300 400 500
b
SS18(QA) -SSX1-EGFP
SS18(YA) -SSX1-EGFP
SS18(GA) -SSX1-EGFP
SS18(PA) -SSX1-EGFP
EGFP
EGFP/DNA
5μm
c
SS18-SSX-HA
SS18(YA)-SSX-HA
H2AK119ub
SS18-SSX binding sites (n=20879)
-5.0 S E -5.0 Kb
d
CUT&Tag
SS18-SSX binding site
H2AK119ub
HA
SS18-SSX
SS18(YA)-SSX
NKX3-2
BOD1L1
PAX6
e
PAX6
SOX2
NKX3-2
Relative expression to Gapdh(%)
Ctr
SS
SS18
SS(QA)
SS(YA)
SS(GA)
SS(PA)
f
EGFP
SS18-SSX1
SS(QA)
SS(YA)
SS(GA)
SS(PA)
Hoechst33342 Azide594
Merge
200μm
g
EdU-positive cells(%)
p=0.0002
p=0.0125
p=0.1093
p=0.0015
p<0.0001
GFP
SS
SS(QA)
SS(YA)
SS(GA)
SS(PA)
h
EGFP
SS18-SSX1
SS(QA)
SS(YA)
SS(GA)
SS(PA)
i
Number of colonies/well
P<0.0001
P<0.0001
P=0.2370
P<0.0001
P<0.0001
j
Control
SS18-SSX1
SS18(YA)-SSX1
Tumor volume (mm³)
p<0.0001
Time after tumour cell inoculation (days)
k
Control
SS18-SSX1
SS18(YA)-SSX1
Tumor weight (g)
p<0.001
l
Control
SS18-SSX1
SS18(YA)-SSX1
Body weight (g)
p=0.2220
ns
Time after tumour cell inoculation (days)

Supplementary Fig. 3
Condensate deficiency mutant SS18(YA)-SSX.

a. Schematic illustration shows the amino acid distribution of SS18-SSX1. The pink box highlights the tyrosine enriched region in SS18 IDR.
b. Representative images for BJ fibroblasts expressing SS18-SSX-EGFP fusion with various IDR mutant via lentiviral infection. Scale bars, 5μm. All the enriched four amino acids Q, Y, G, P in SS18 IDR were mutated by alanine (QA, YA, GA, PA), respectively.
c. Heatmap shows the intensity of SS18(YA)-SSX mutant and H2AK119ub occupancy at SS18-SSX binding sites in BJ cells.
d. Representative genome view of H2AK119ub, SS18-SSX and SS18(YA)-SSX occupancy at the typical downstream genes of SS18-SSX.
e. The expression of representative downstream genes of SS18-SSX in BJ fibroblasts with lentiviral expression of EGFP, SS18, SS18-SSX and the corresponding four mutants of SS18(QA)-SSX, SS18(YA)-SSX, SS18(GA)-SSX and SS18(PA)-SSX, respectively. Data are mean±s.d., n=3 independent experiments.
f. Representative image of EdU assay in synovial sarcoma cell line SW982 with lentiviral expression of EGFP, wildtype and four mutants of SS18-SSX1. Scale bars, 200μm.
g. Histogram shows the ratio of EdU positive cells in (f). Data are mean±s.d., two-sided, unpaired t-test, *$p < 0.05$, **$p < 0.01$, ***$p < 0.001$, ****$p < 0.0001$, ns, not significant. n= 3 independent experiments.
h. Representative image of colony formation assay in synovial sarcoma cell line SW982 overexpressing EGFP, wildtype and four mutants of SS18-SSX1, respectively. One well of a 6-well plate was seeded with 1,000 cells and cultured for 10 days. Colonies were stained with 0.1% crystal violet.
i. Histogram shows the number of colonies in one well of (h). Data are mean±s.d., two-sided, unpaired t-test, ****$p < 0.0001$, ns, not significant. n= 6, from 3 independent experiments.
j. Tumor growth curve of SW982 cell overexpressing EGFP, SS18-SSX and SS18(YA)-SSX xenografts established in NCG mice for 21 days. Data are

mean ± s.d., two-sided, unpaired t test of n = 4 mice per group from two biological replicates, ****p < 0.0001.

k. Left panel shows the xenografts 21 days in (j) (length unit: centimeter). The right panel shows the corresponding weight in one representative experiment. Data are mean ± s.d., two-sided, unpaired t test of n = 4 mice per group from two biological replicates, **** p < 0.0001.

l. Body weights dynamics of mice in (j). Data are mean ± s.d., twosided, unpaired t test of n = 4 mice per group, ns, not significant.

Figure S4

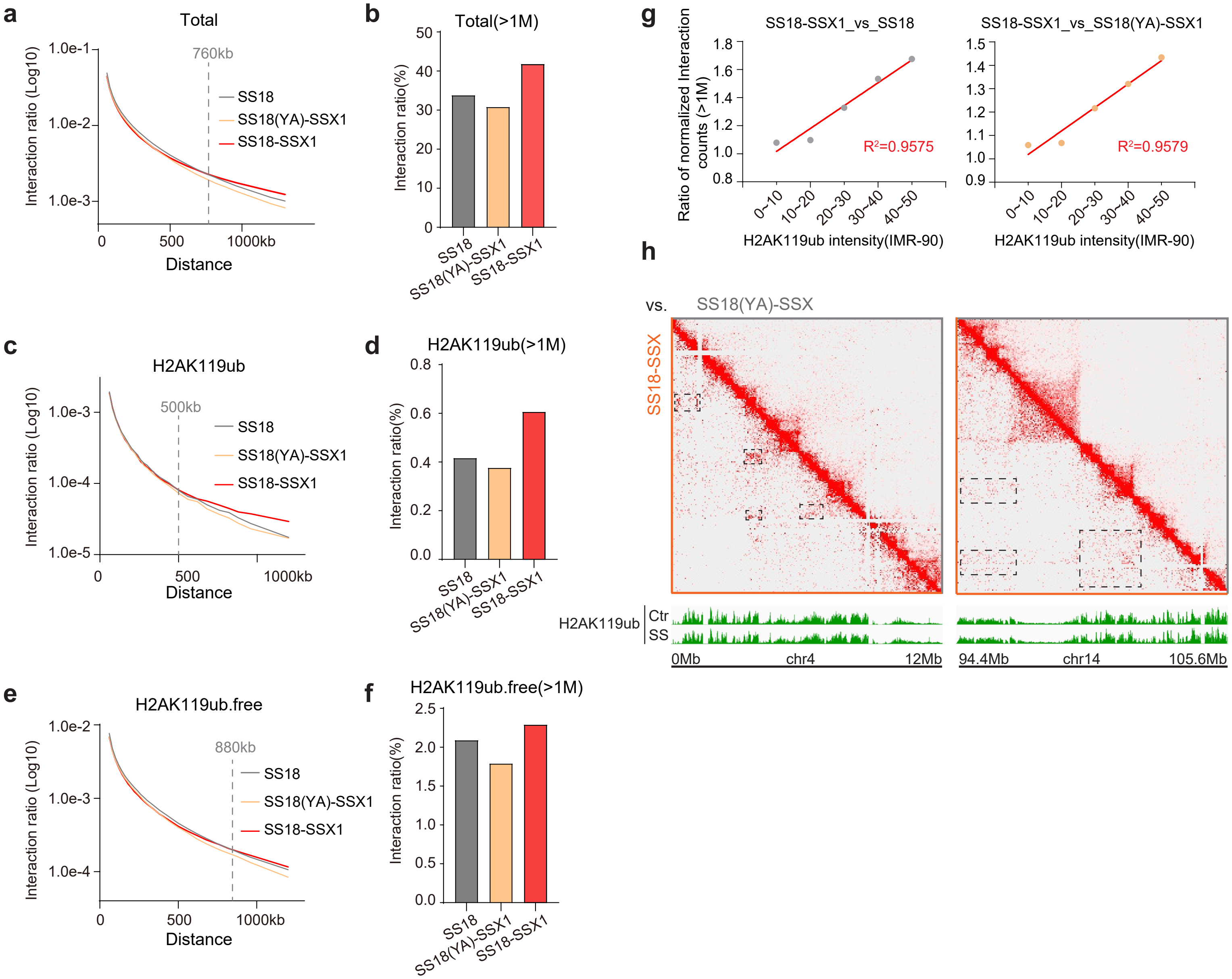

a
Total
760kb
SS18
SS18(YA)-SSX1
SS18-SSX1
Interaction ratio (Log10)
Distance
b
Total(>1M)
Interaction ratio(%)
c
H2AK119ub
500kb
d
H2AK119ub(>1M)
e
H2AK119ub.free
880kb
f
H2AK119ub.free(>1M)
g
SS18-SSX1_vs_SS18
SS18-SSX1_vs_SS18(YA)-SSX1
Ratio of normalized Interaction counts (>1M)
R²=0.9575
R²=0.9579
H2AK119ub intensity(IMR-90)
h
vs. SS18(YA)-SSX
SS18-SSX
H2AK119ub
Ctr
SS
0Mb
chr4
12Mb
94.4Mb
chr14
105.6Mb

Supplementary Fig. 4

SS18-SSX condensates promote the long-range chromatin interaction.

a. The curves show the distribution of whole genome chromatin interaction ratio at different distance within cells with expression of SS18, SS18(YA)-SSX mutant and SS18-SSX, respectively.

b. The histogram counts the ratio of chromatin interaction in (a) at the distance of more than 1 Mb.

c. The curves show the distribution of the interaction ratio of the chromatin with H2AK119ub modification at different distance within cells with expression of SS18, SS18(YA)-SSX mutant and SS18-SSX, respectively.

d. The histogram counts the ratio of chromatin interaction in (c) at the distance of more than 1 Mb.

e. The curves show the distribution of the interaction ratio of the chromatin without H2AK119ub modification at different distance within cells with expression of SS18, SS18(YA)-SSX mutant and SS18-SSX, respectively.

f. The histogram counts the ratio of chromatin interaction in (e) at the distance of more than 1 Mb.

g. Correlation analysis between the H2AK119ub intensity and the enhance ratio of long-range (more than 1Mb) chromatin interaction comparing SS18-SSX with wildtype SS18 (the left) or condensate deficiency mutant SS18(YA)-SSX (the right) in IMR-90 cells.

h. The representative regions at chromosome 4 and 14 show the correlation of enhanced long-range chromatin interaction with H2AK119ub modification in IMR-90 cells.

Figure S5

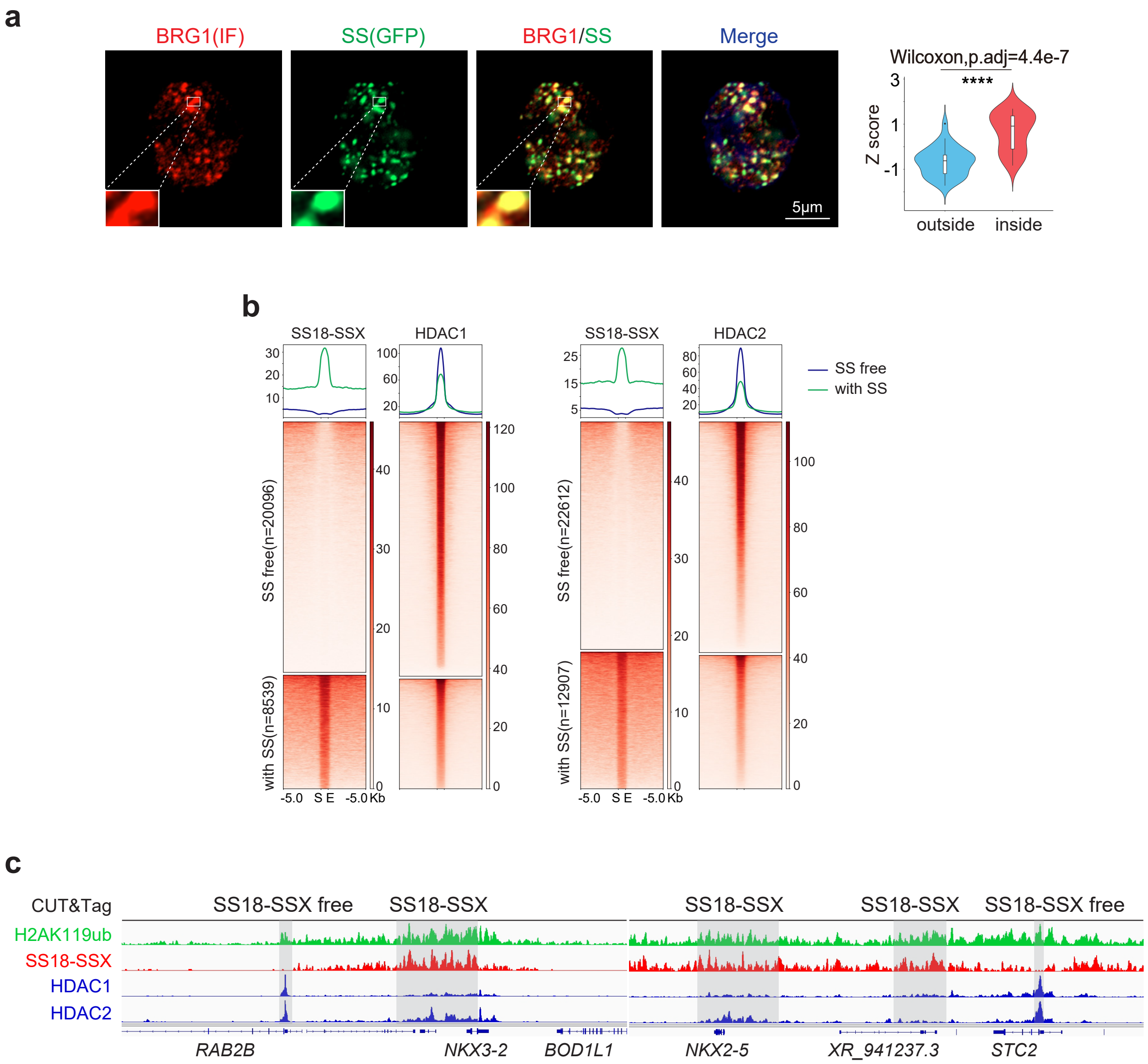

a
BRG1(IF)
SS(GFP)
BRG1/SS
Merge
5μm
Wilcoxon,p.adj=4.4e-7
****
Z score
outside
inside
b
SS18-SSX
HDAC1
SS18-SSX
HDAC2
SS free
with SS
SS free(n=20096)
with SS(n=8539)
SS free(n=22612)
with SS(n=12907)
-5.0 S E -5.0 Kb
c
CUT&Tag
SS18-SSX free
SS18-SSX
H2AK119ub
SS18-SSX
HDAC1
HDAC2
RAB2B
NKX3-2
BOD1L1
NKX2-5
XR_941237.3
STC2

Supplementary Fig. 5

The correlation between SS18-SSX and BRG1 or HDACs.

a. On the left panels, representative images of immunofluorescence of BRG1 in HEK293T cells expressing SS18-SSX1-EGFP protein. Scale bar, 5 μm. The violin plot on the right panel shows the quantitative analysis of co-localization. Outside and inside groups indicate the distribution of random pixels' fluorescence intensity of BRG1 normalized by Z score from outside and inside of SS18-SSX condensates. Two-sided Wilcoxon test adjusted for multiple comparisons. n = 30 pixels, from 3 nuclei. ****p < 0.0001. ns, not significant. SS, SS18-SSX1.

b. The heatmap shows the differential binding intensity of HDAC1/2 at the sites with or without colocalization with SS18-SSX.

c. Representative genome views show the relationship between the binding intensity of HDAC1/2 and SS18-SSX.

Figure S6

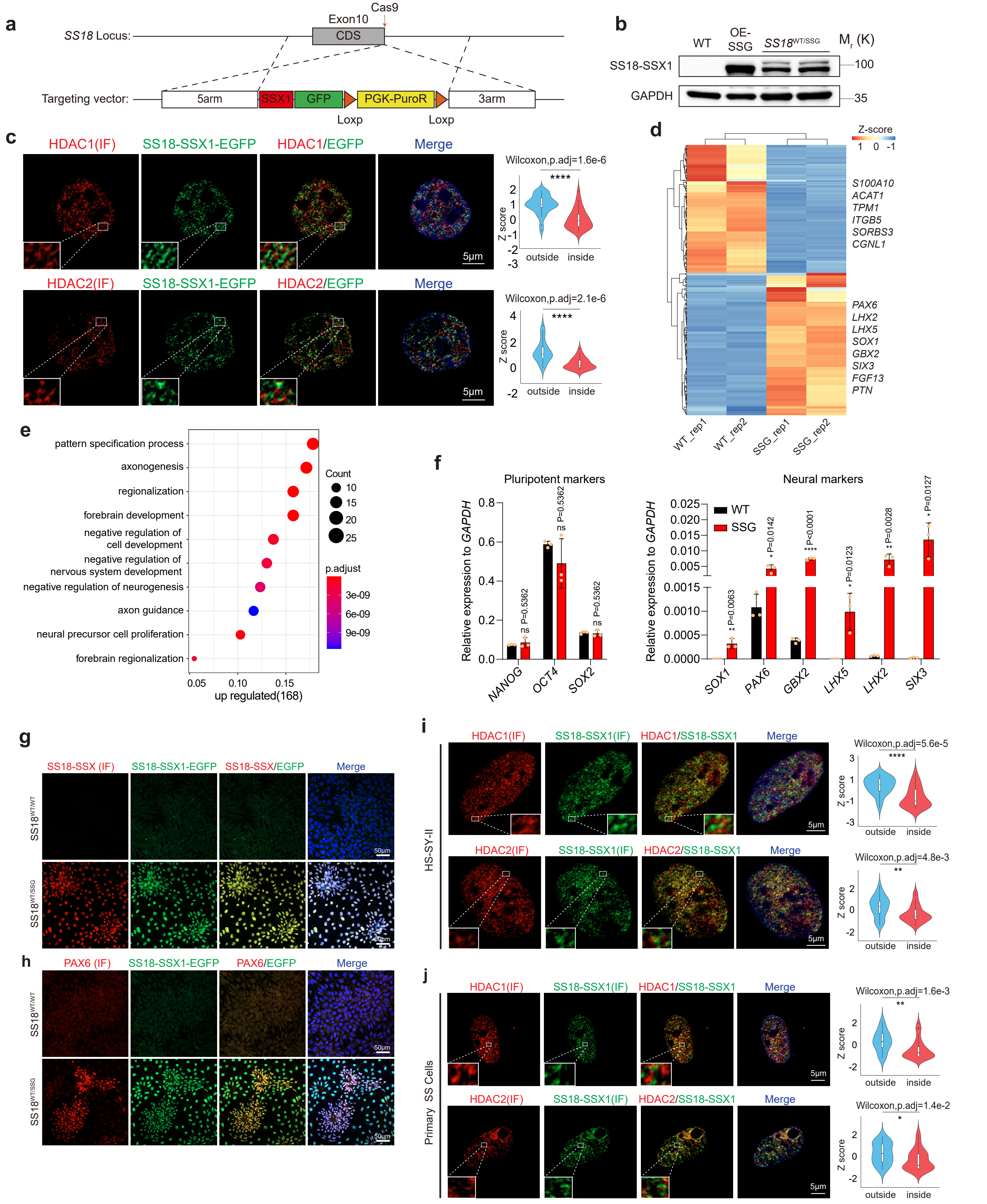
a
SS18 Locus:
Exon10
Cas9
CDS
Targeting vector:
5arm
SSX1
GFP
PGK-PuroR
3arm
Loxp
Loxp
b
WT
OE-SSG
SS18WT/SSG
Mr (K)
SS18-SSX1
GAPDH
100
35
c
HDAC1(IF)
SS18-SSX1-EGFP
HDAC1/EGFP
Merge
Wilcoxon,p.adj=1.6e-6
HDAC2(IF)
HDAC2/EGFP
Wilcoxon,p.adj=2.1e-6
Z score
outside
inside
5μm
d
Z-score
S100A10
ACAT1
TPM1
ITGB5
SORBS3
CGNL1
PAX6
LHX2
LHX5
SOX1
GBX2
SIX3
FGF13
PTN
WT_rep1
WT_rep2
SSG_rep1
SSG_rep2
e
pattern specification process
axonogenesis
regionalization
forebrain development
negative regulation of cell development
negative regulation of nervous system development
negative regulation of neurogenesis
axon guidance
neural precursor cell proliferation
forebrain regionalization
Count
p.adjust
up regulated(168)
f
Pluripotent markers
Neural markers
Relative expression to GAPDH
WT
SSG
NANOG
OCT4
SOX2
SOX1
PAX6
GBX2
LHX5
LHX2
SIX3
P=0.5362
P=0.0063
P=0.0142
P<0.0001
P=0.0123
P=0.0028
P=0.0127
g
SS18-SSX (IF)
SS18-SSX1-EGFP
SS18-SSX/EGFP
Merge
SS18WT/WT
SS18WT/SSG
50μm
h
PAX6 (IF)
PAX6/EGFP
i
HS-SY-II
SS18-SSX1(IF)
HDAC1/SS18-SSX1
HDAC2/SS18-SSX1
Wilcoxon,p.adj=5.6e-5
Wilcoxon,p.adj=4.8e-3
j
Primary SS Cells
Wilcoxon,p.adj=1.6e-3
Wilcoxon,p.adj=1.4e-2

Supplementary Fig. 6

The endogenous SS18-SSX condensates exclude HDACs.

a. Schematic illustration shows the strategy to mimic the endogenous expression of SS18-SSX1-EGFP by knocking in C-SSX1-EGFP at the SS18 locus in human stem cells (hESCs).

b. The expression of SS18-SSX1-EGFP were confirmed by western blotting.

c. Representative immunofluorescent images of endogenous HDAC1/2 and endogenous SS18-SSX1-EGFP on the left panels. Scale bars, 5μm. The violin plots on the right panels show the quantitative analysis of co-localization. Outside and inside groups indicate the distribution of random pixels' fluorescence intensity of HDAC1/2 normalized by Z score from outside and inside of SS18-SSX1-EGFP condensates. Two-sided Wilcoxon test adjusted for multiple comparisons. n = 30 pixels, from 3 nuclei. ****p < 0.0001.

d. Heat map showing the differentially expressed genes between wild type and endogenous SS18-SSX expression human stem cells.

e. Gene ontology analysis indicated the most significantly up-regulated genes sets upon endogenous expression of SS18-SSX in hESCs. One-sided hypergeometric test adjusted for multiple comparisons.

f. The expression of representative pluripotent and neural genes in hESCs with expression of SS18-SSX-EGFP endogenously. Data are mean±s.d., n=3 independent experiments. *p < 0.05, **p < 0.01, ****p < 0.0001.

g. Representative immunofluorescent images of SS18-SSX in hESCs with expression of SS18-SSX-EGFP endogenously. Scale bars, 50μm.

h. Representative immunofluorescent images of endogenous PAX6 in hESCs with expression of SS18-SSX-EGFP endogenously. Scale bars, 50μm.

i. Representative immunofluorescent images of endogenous HDAC1/2 and SS18-SSX in HS-SY-II synovial sarcoma cells on the left panels. Scale bars, 5μm. The violin plots on the right panels show the quantitative analysis of co-localization. Outside and inside groups indicate the distribution of

random pixels' fluorescence intensity of HDAC1/2 normalized by Z score from outside and inside of SS18-SSX condensates. Two-sided Wilcoxon test adjusted for multiple comparisons. n = 30 pixels, from 3 nuclei. $^{**}p < 0.01$, $^{****}p < 0.0001$.

j. Representative immunofluorescent images of endogenous HDAC1/2 and SS18-SSX in primary synovial sarcoma cells on the left panels. Scale bars, 5μm. The violin plots on the right panels show the quantitative analysis of co-localization. Outside and inside groups indicate the distribution of random pixels' fluorescence intensity of HDAC1/2 normalized by Z score from outside and inside of SS18-SSX condensates. Two-sided Wilcoxon test adjusted for multiple comparisons. n = 30 pixels, from 3 nuclei. $^{*}p < 0.05$, $^{**}p < 0.01$.

Figure S7

a

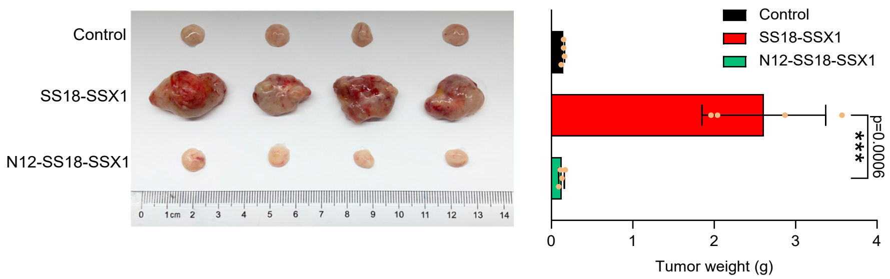
Control
SS18-SSX1
N12-SS18-SSX1
Control
SS18-SSX1
N12-SS18-SSX1
***
p=0.0006
0
1
2
3
4
Tumor weight (g)

b

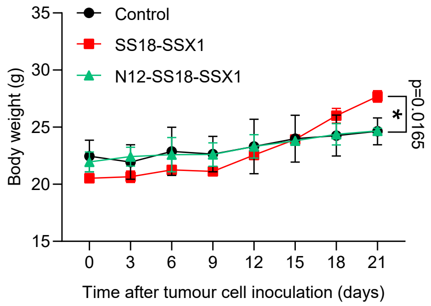
Control
SS18-SSX1
N12-SS18-SSX1
35
30
25
20
15
Body weight (g)
*
p=0.0165
0
3
6
9
12
15
18
21
Time after tumour cell inoculation (days)

Supplementary Fig. 7

N12 domain impedes tumorigenesis in tumor-bearing mouse models

a. Left panel shows the xenografts 21 days in (Fig 3h). Length unit: centimeter. The right panel shows the corresponding weight in one representative experiment. Data are mean ± s.d., two-sided, unpaired t test of $n = 4$ mice per group from two biological replicates, *** $p < 0.001$.

b. Body weights dynamics of mice in (Fig 3h). Data are mean ± s.d., twosided, unpaired t test of $n = 4$ mice per group, * $p < 0.05$.

Figure S8

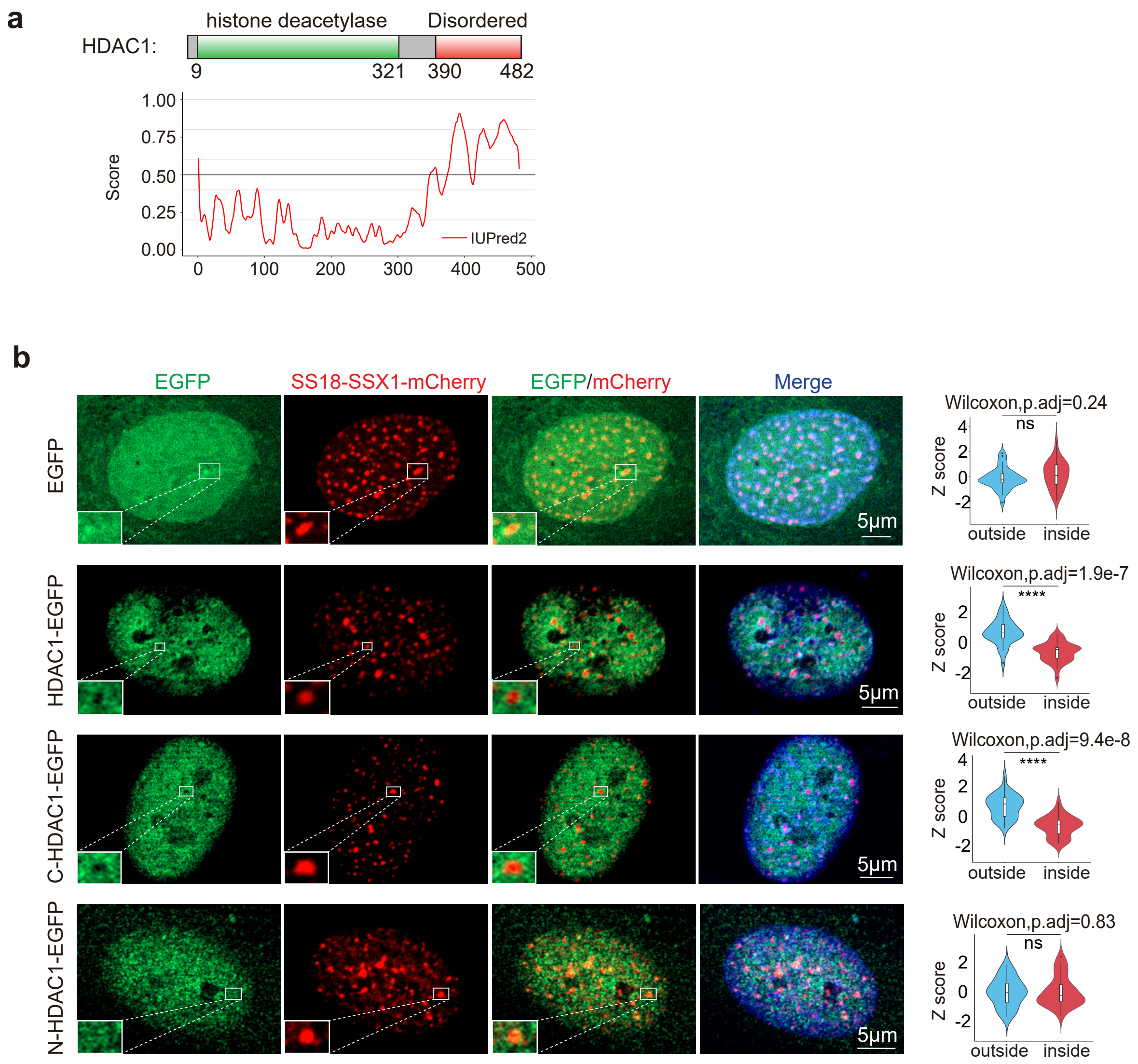
a
histone deacetylase
Disordered
HDAC1:
9
321
390
482
Score
1.00
0.75
0.50
0.25
0.00
0
100
200
300
400
500
IUPred2
b
EGFP
SS18-SSX1-mCherry
EGFP/mCherry
Merge
EGFP
HDAC1-EGFP
C-HDAC1-EGFP
N-HDAC1-EGFP
5μm
Wilcoxon,p.adj=0.24
ns
Wilcoxon,p.adj=1.9e-7
****
Wilcoxon,p.adj=9.4e-8
****
Wilcoxon,p.adj=0.83
ns
Z score
outside
inside

Supplementary Fig. 8

The C-terminal region of HDAC1 was excluded by SS18-SSX condensates.

a. Schematic illustration of HDAC1 protein containing a N-terminal deacetylase domain and a C-terminal disordered domain (upper panel) and graph plotting intrinsic disorder of HDAC1 by the IUPred2A algorithm (https://iupred2a.elte.hu/). IUPred2 scores are shown on the y axis, and the amino acid positions are shown on the x axis (lower panel).

b. Representative image of BJ fibroblasts with lentiviral co-expression of SS18-SSX1-mCherry and EGFP, HDAC1-EGFP, EGFP fused C or N terminus of HDAC1 on the left panel. Scale bars, 5μm. Outside and inside groups indicate the distribution of random pixels' fluorescence intensity of EGFP normalized by Z score from outside and inside of SS18-SSX1 condensates, respectively. Two-sided Wilcoxon test adjusted for multiple comparisons. n = 30 pixels, from 3 nuclei. ****$p < 0.0001$. ns, not significant.

Figure S9

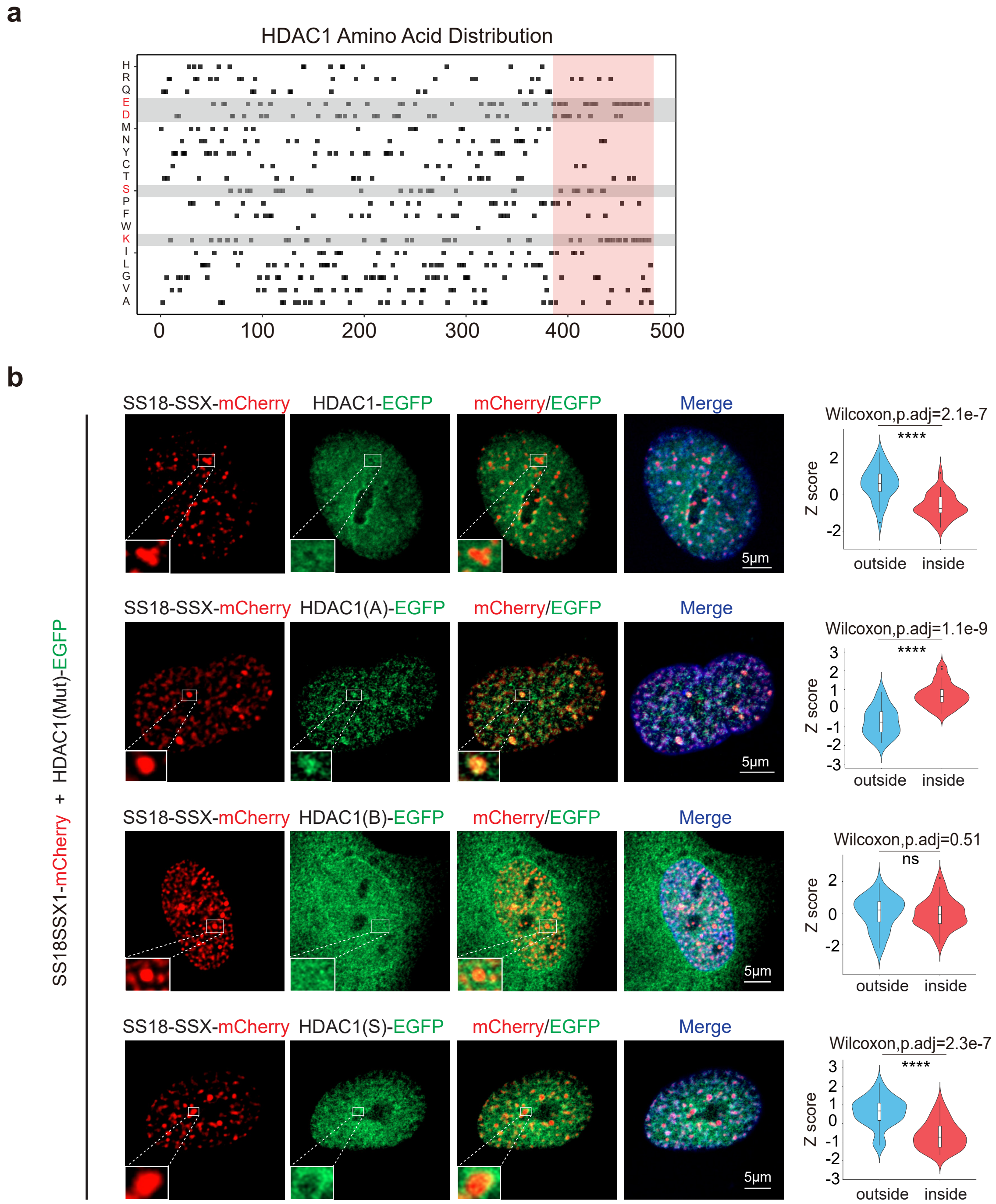
a
HDAC1 Amino Acid Distribution
H
R
Q
E
D
M
N
Y
C
T
S
P
F
W
K
I
L
G
V
A
0
100
200
300
400
500
b
SS18SSX1-mCherry + HDAC1(Mut)-EGFP
SS18-SSX-mCherry
HDAC1-EGFP
mCherry/EGFP
Merge
5μm
Wilcoxon,p.adj=2.1e-7
****
Z score
outside
inside
SS18-SSX-mCherry
HDAC1(A)-EGFP
mCherry/EGFP
Merge
5μm
Wilcoxon,p.adj=1.1e-9
****
Z score
outside
inside
SS18-SSX-mCherry
HDAC1(B)-EGFP
mCherry/EGFP
Merge
5μm
Wilcoxon,p.adj=0.51
ns
Z score
outside
inside
SS18-SSX-mCherry
HDAC1(S)-EGFP
mCherry/EGFP
Merge
5μm
Wilcoxon,p.adj=2.3e-7
****
Z score
outside
inside

Supplementary Fig. 9

The electrical property of IDR regulates HDAC1 exclusion.

a. Schematic illustration shows the amino acid distribution of HDAC1. The pink box highlights the C-terminal disordered region of HDAC1.

b. Representative image (left panel) of BJ fibroblasts with lentiviral co-expression of SS18-SSX1-mCherry and EGFP fused HDAC1 mutation. A, B and S indicated all the acidic amino acids, basic amino acids and serine from the pink box in (a) were mutated to alanine, respectively. Scale bars, 5μm. The violin plots on the right panels show the quantitative analysis of co-localization. Outside and inside groups indicate the distribution of random pixels' fluorescence intensity of EGFP normalized by Z score from outside and inside of SS18-SSX condensates, respectively. Two-sided Wilcoxon test adjusted for multiple comparisons. n = 30 pixels, from 3 nuclei. ****p < 0.0001. ns, not significant.

Figure S10

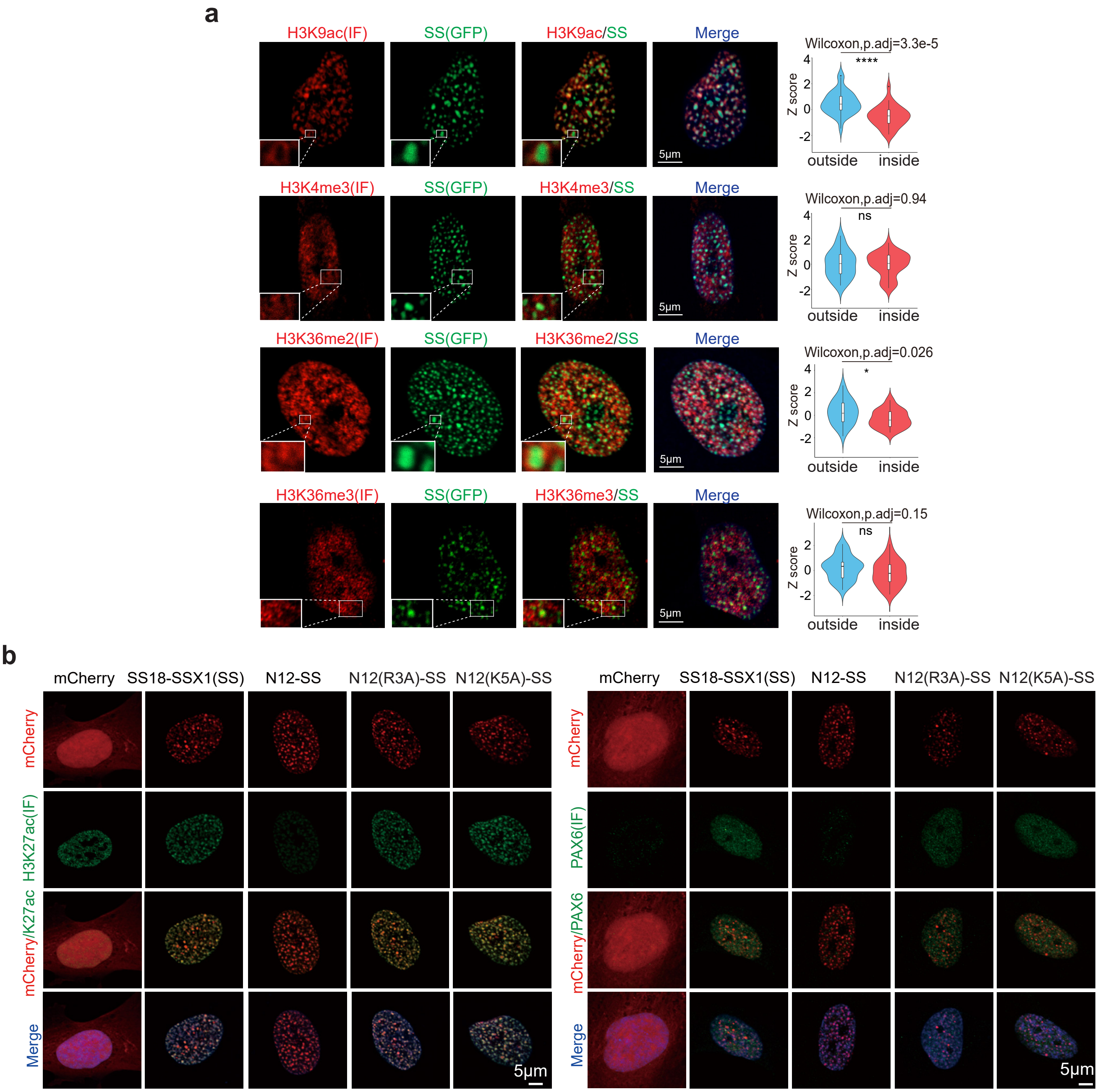
a
H3K9ac(IF)
SS(GFP)
H3K9ac/SS
Merge
5μm
Wilcoxon,p.adj=3.3e-5
****
Z score
outside
inside
H3K4me3(IF)
SS(GFP)
H3K4me3/SS
Merge
5μm
Wilcoxon,p.adj=0.94
ns
Z score
outside
inside
H3K36me2(IF)
SS(GFP)
H3K36me2/SS
Merge
5μm
Wilcoxon,p.adj=0.026
*
Z score
outside
inside
H3K36me3(IF)
SS(GFP)
H3K36me3/SS
Merge
5μm
Wilcoxon,p.adj=0.15
ns
Z score
outside
inside
b
mCherry
SS18-SSX1(SS)
N12-SS
N12(R3A)-SS
N12(K5A)-SS
mCherry
H3K27ac(IF)
mCherry/K27ac
Merge
5μm
mCherry
SS18-SSX1(SS)
N12-SS
N12(R3A)-SS
N12(K5A)-SS
mCherry
PAX6(IF)
mCherry/PAX6
Merge
5μm

Supplementary Fig. 10

H3K27ac in the BJ fibroblasts expressing onco-fusion.

a. Representative image (left panel) of immunofluorescence for H3K9ac 、H3K4me3、H3K36me2 and H3K36me3 in the BJ fibroblasts with lentiviral expression of SS18-SSX1-EGFP. Scale bars, 5μm. The violin plots on the right panels show the quantitative analysis of co-localization. Outside and inside groups indicate the distribution of random pixels' fluorescence intensity of H3K9ac 、H3K4me3、H3K36me2 and H3K36me3 normalized by Z score from outside and inside of SS18-SSX-EGFP condensates, respectively. Two-sided Wilcoxon test adjusted for multiple comparisons. n = 30 pixels, from 3 nuclei. *$p < 0.05$, ****$p < 0.0001$. ns, not significant.

b. Representative image of immunofluorescence for H3K27ac and PAX6 in the BJ fibroblasts with lentiviral expression of SS18-SSX1, SALLs derived N12 domain tagged SS18-SSX1, R3A or K5A mutated N12 tagged SS18-SSX1, respectively. SS, SS18-SSX1. Scale bars, 5μm.

Figure S11

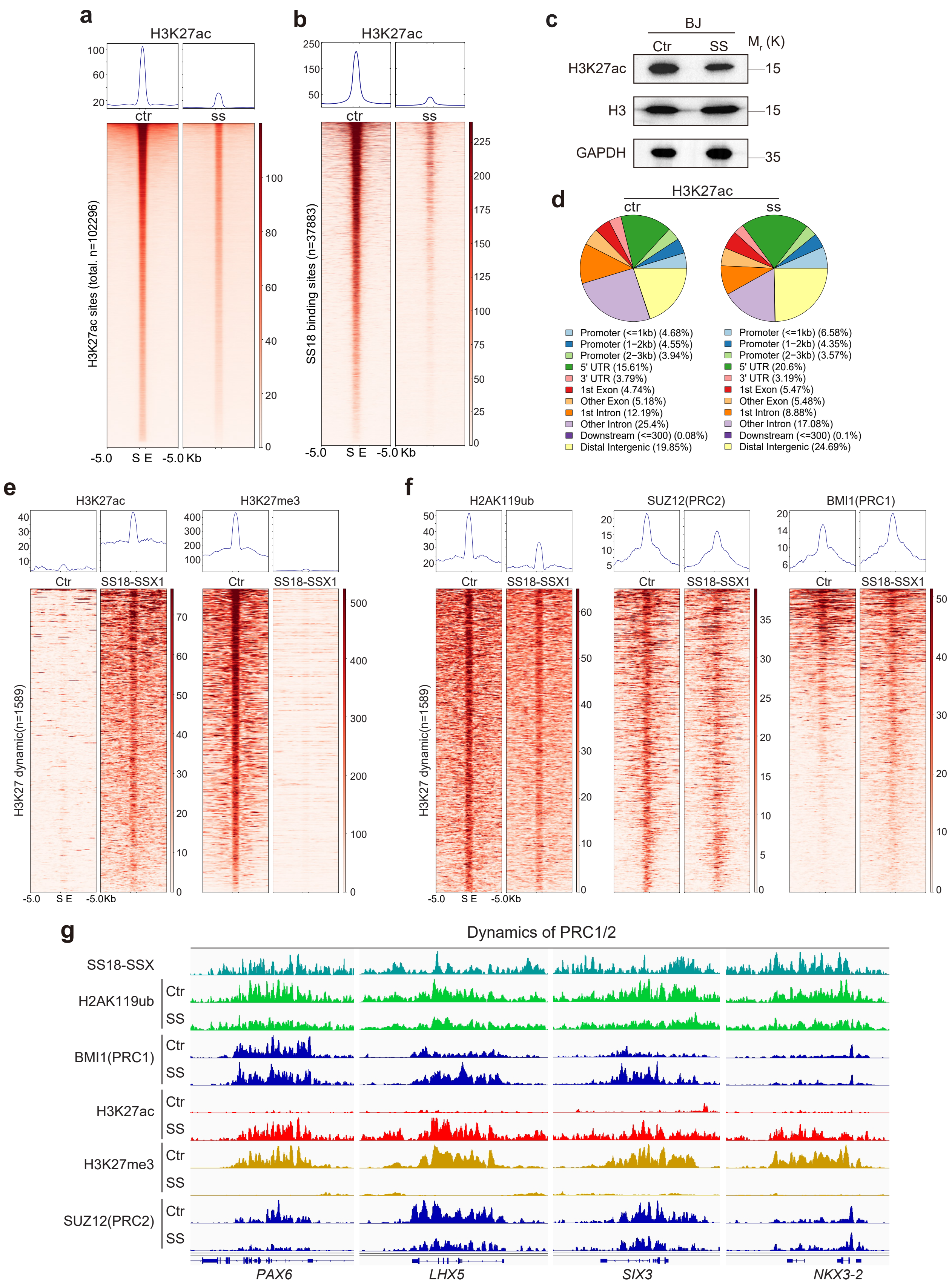

a
H3K27ac
ctr
ss
H3K27ac sites (total. n=102296)
-5.0 S E -5.0 Kb
b
H3K27ac
ctr
ss
SS18 binding sites (n=37883)
-5.0 S E -5.0 Kb
c
BJ
Ctr
SS
Mr (K)
H3K27ac
H3
GAPDH
15
15
35
d
H3K27ac
ctr
ss
Promoter (<=1kb) (4.68%)
Promoter (1−2kb) (4.55%)
Promoter (2−3kb) (3.94%)
5' UTR (15.61%)
3' UTR (3.79%)
1st Exon (4.74%)
Other Exon (5.18%)
1st Intron (12.19%)
Other Intron (25.4%)
Downstream (<=300) (0.08%)
Distal Intergenic (19.85%)
Promoter (<=1kb) (6.58%)
Promoter (1−2kb) (4.35%)
Promoter (2−3kb) (3.57%)
5' UTR (20.6%)
3' UTR (3.19%)
1st Exon (5.47%)
Other Exon (5.48%)
1st Intron (8.88%)
Other Intron (17.08%)
Downstream (<=300) (0.1%)
Distal Intergenic (24.69%)
e
H3K27ac
H3K27me3
Ctr
SS18-SSX1
H3K27 dynamic(n=1589)
-5.0 S E -5.0Kb
f
H2AK119ub
SUZ12(PRC2)
BMI1(PRC1)
Ctr
SS18-SSX1
H3K27 dynamic(n=1589)
-5.0 S E -5.0Kb
g
Dynamics of PRC1/2
SS18-SSX
H2AK119ub
BMI1(PRC1)
H3K27ac
H3K27me3
SUZ12(PRC2)
Ctr
SS
PAX6
LHX5
SIX3
NKX3-2

Supplementary Fig. 11

The abnormal epigenetic landscape upon SS18-SSX expression.

a. The heatmap shows the intensity of H3K27ac histone modification occupation at total H3K27ac sites in BJ fibroblasts with or without expression of SS18-SSX1 onco-fusion.

b. The heatmap shows the intensity of H3K27ac histone modification occupation at SS18 binding sites in BJ fibroblasts with or without expression of SS18-SSX1 onco-fusion.

c. The expression level of H3K27ac were confirmed by western blotting in BJ fibroblasts with or without expression of SS18-SSX1 onco-fusion.

d. Pie charts show the dynamic distribution of the indicated genomic annotation feature among the H3K27ac sites in BJ cells before and after SS18-SSX expression.

e. Heatmap shows the 1589 genomic sites with a significant H3K27ac/me3 dynamic change.

f. The heatmaps show the intensity changes of H2AK119ub, PRC1(BMI1) and PRC2(SUZ12) occupancy at the genomic sites of (f).

g. Representative genomic views show the epigenomic landscape at the sites with H3K27ac/me3 dynamic changes.

Figure S12

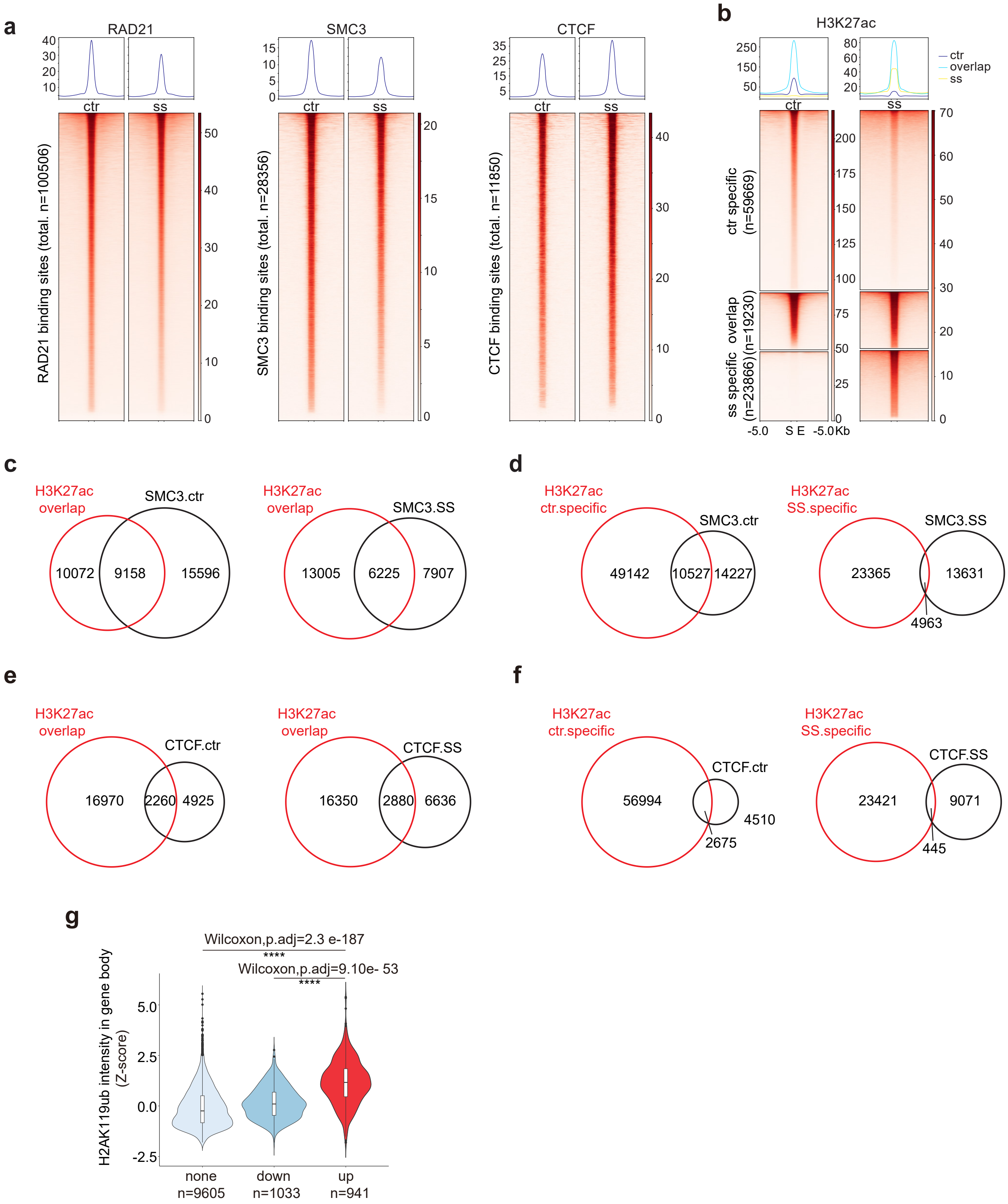

a
RAD21
SMC3
CTCF
ctr
ss
RAD21 binding sites (total. n=100506)
SMC3 binding sites (total. n=28356)
CTCF binding sites (total. n=11850)
b
H3K27ac
ctr
overlap
ss
ctr specific (n=59669)
overlap (n=19230)
ss specific (n=23866)
-5.0
S E
-5.0Kb
c
H3K27ac overlap
SMC3.ctr
10072
9158
15596
SMC3.SS
13005
6225
7907
d
H3K27ac ctr.specific
49142
10527
14227
H3K27ac SS.specific
SMC3.SS
23365
13631
4963
e
CTCF.ctr
16970
2260
4925
CTCF.SS
16350
2880
6636
f
56994
4510
2675
23421
9071
445
g
Wilcoxon,p.adj=2.3 e-187
****
Wilcoxon,p.adj=9.10e- 53
****
H2AK119ub intensity in gene body (Z-score)
none n=9605
down n=1033
up n=941

Supplementary Fig. 12

Joint analysis of H3K27ac and cohesin-CTCF

a. Heatmaps show the dynamic changes of the occupancy intensity of cohesin-CTCF at the total binding sites in BJ cells with the overexpression of SS18-SSX1 and SS18(YA)-SSX as control. SS, SS18-SSX.

b. The heatmaps show the total H3K27ac sites in BJ cells with the overexpression of SS18-SSX1 and SS18(YA)-SSX as control were divided into three groups, i.e., control specific sites, SS18-SSX1 specific sites and the overlapped sites. SS, SS18-SSX.

c. The colocalization analysis between the overlapped H3K27ac sites in (b) with cohesin component SMC3 binding sites in BJ cells with the overexpression of SS18-SSX1 and SS18(YA)-SSX as control, respectively. SS, SS18-SSX1.

d. The colocalization analysis between the specific H3K27ac sites in (b) with cohesin component SMC3 binding sites in BJ cells with the overexpression of SS18-SSX1 and SS18(YA)-SSX as control, respectively. SS, SS18-SSX1.

e. The colocalization analysis between the overlapped H3K27ac sites in (b) with CTCF binding sites in BJ cells with the overexpression of SS18-SSX1 and SS18(YA)-SSX as control, respectively. SS, SS18-SSX1.

f. The colocalization analysis between the specific H3K27ac sites in (b) with CTCF binding sites in BJ cells with the overexpression of SS18-SSX1 and SS18(YA)-SSX as control, respectively. SS, SS18-SSX1.

g. Violin plot shows the differential deposition of H2AK119ub histone modification on the gene body of distinct expression regulatory types. Two-sided Wilcoxon test adjusted for multiple comparisons. **** $p < 0.0001$.

Figure S13

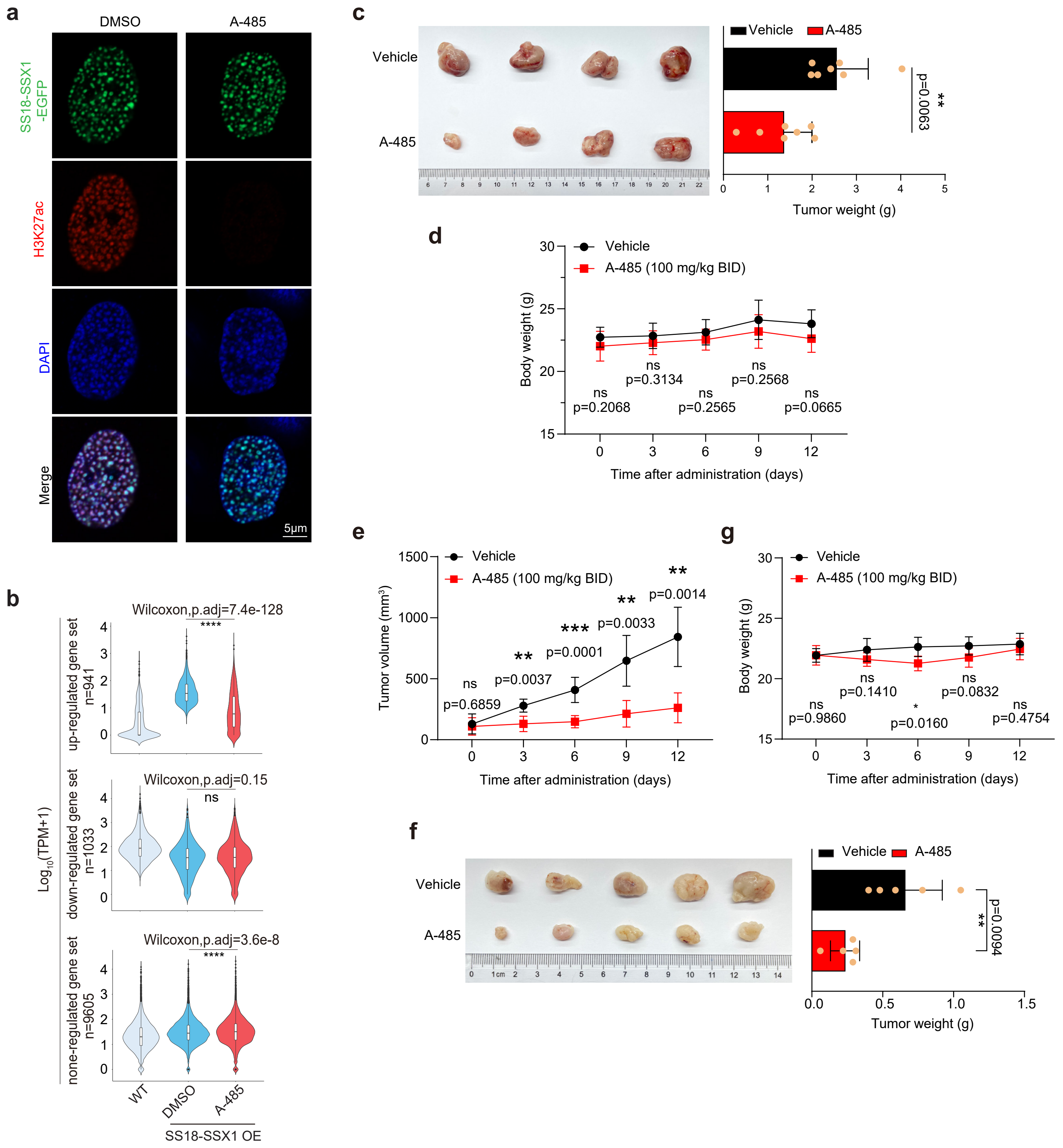
a
DMSO
A-485
SS18-SSX1 -EGFP
H3K27ac
DAPI
Merge
5μm
b
Wilcoxon,p.adj=7.4e-128
****
up-regulated gene set n=941
Wilcoxon,p.adj=0.15
ns
down-regulated gene set n=1033
Wilcoxon,p.adj=3.6e-8
****
none-regulated gene set n=9605
Log10(TPM+1)
WT
DMSO
A-485
SS18-SSX1 OE
c
Vehicle
A-485
Tumor weight (g)
p=0.0063
**
d
Vehicle
A-485 (100 mg/kg BID)
Body weight (g)
ns p=0.2068
ns p=0.3134
ns p=0.2565
ns p=0.2568
ns p=0.0665
Time after administration (days)
e
Vehicle
A-485 (100 mg/kg BID)
Tumor volume (mm³)
ns p=0.6859
** p=0.0037
*** p=0.0001
** p=0.0033
** p=0.0014
Time after administration (days)
g
Vehicle
A-485 (100 mg/kg BID)
Body weight (g)
ns p=0.9860
ns p=0.1410
* p=0.0160
ns p=0.0832
ns p=0.4754
Time after administration (days)
f
Vehicle
A-485
Tumor weight (g)
p=0.0094
**

Supplementary Fig. 13

A-485 constrains tumor growth in safe doses.

a. The representative image of immunofluorescence for H3K27ac in SS18-SSX1-EGFP expressed BJ fibroblasts with or without treatment of 10μM A-485 inhibitor for 3h. Scale bars, 5μm.

b. Violin plot showing the changes of gene expression upon treatment with 1μM A-485 for 24h in BJ fibroblasts expressing SS18-SSX1 fusion. The upper, middle and lower panel represent the expressing changes of up-regulated, downregulated and none-regulated gene set of SS18-SSX1, respectively. Two-sided Wilcoxon test adjusted for multiple comparisons. ****$p < 0.0001$. ns, not significant.

c. Xenografts treated with either vehicle control or A-485 for 12 days from (Figure 5e) in one representative experiment (left panel, length unit: centimeter). The right panel shows the corresponding weight. Data are mean ± s.d., two-sided, unpaired t test of $n = 7$ mice per group from two biological replicates. ** $p < 0.01$.

d. Body weights dynamics of mice from (Figure 5e). Data are mean ± s.d., two-sided, unpaired t test of $n = 7$ mice per group, ns, not significant.

e. Tumor growth curve of HS-SY-II cell xenografts established in NCG mice. Mice were treated intraperitoneally with vehicle control or A-485 at 100 mg/kg/dose, twice daily for 12 days (BID × 12). Data are mean ± s.d., two-sided, unpaired t test of $n = 5$ mice per group from two biological replicates. **$p < 0.01$. ***$p < 0.001$. ns, not significant.

f. Xenografts treated with either vehicle control or A-485 for 12 days from (Figure S13e) (left panel, length unit: centimeter). The right panel shows the corresponding weight. Data are mean ± s.d., two-sided, unpaired t test of $n = 5$ mice per group from two biological replicates. **$p < 0.01$.

g. Body weights dynamics of mice from (Figure S13e). Data are mean ± s.d., two-sided, unpaired t test of $n = 5$ mice per group from two biological replicates. *$p < 0.05$. ns, not significant.
