# Supplementary figures and images for: Exclusion of HDAC1/2 complexes by oncogenic nuclear condensates
Source: Mol Cancer. 2024 Apr 27;23:85. doi: 10.1186/s12943-024-02002-1 (PMC11055323; doi:10.1186/s12943-024-02002-1)

# Original uncropped western blots

Figure 1d

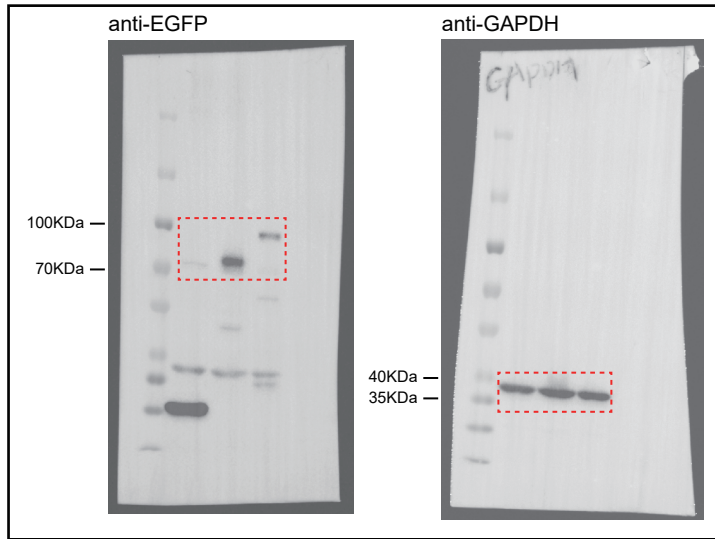

Figure S11c

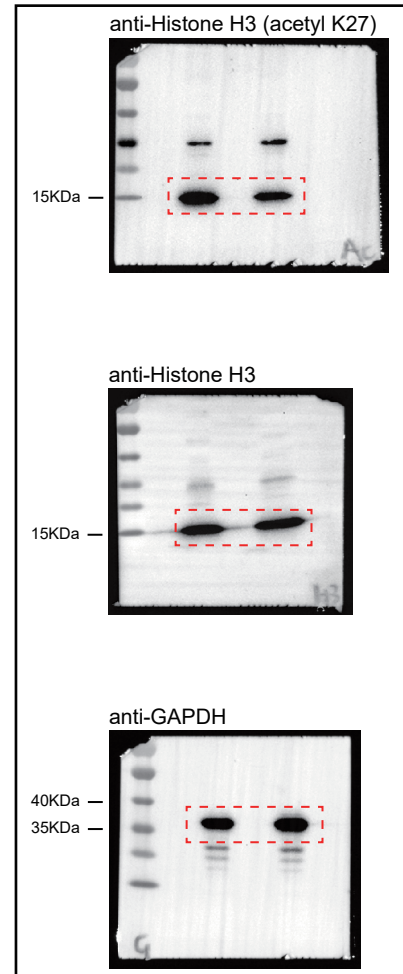

Figure S6b

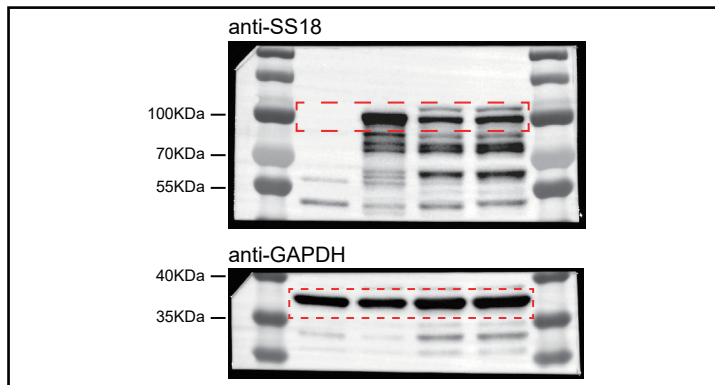

Supplement: Supplementary file 2 — Supplementary Material 2. [file 12943_2024_2002_MOESM2_ESM.pdf]
